# Elucidating the neuromeric organization of the Mongolian gerbil brain

**DOI:** 10.1007/s00429-025-03018-z

**Published:** 2025-11-04

**Authors:** F. Lucero-Arteaga, S. Labegorra, A. Abrego-Alvarez, V. Heck, A. I. Portu, M. A. Boeris, A. Alonso, B. Ribeiro Do-Couto, M. Á. García-Cabezas, K. Y. Tseng, José Luis Ferran

**Affiliations:** 1 Consejo Nacional de Investigaciones Científicas y Técnicas (CONICET), Buenos Aires, Argentina; 2 Centro de Producción de Animales de Experimentación (CePAE), Facultad de Ciencias Veterinarias, Universidad Nacional de La Pampa, General Pico, Argentina; 3 Instituto Murciano de Investigación Biosanitaria Pascual Parrilla–IMIB, Murcia, 30120 España; 4 Department of Human Anatomy and Psychobiology, Faculty of Medicine, University of Murcia, Murcia, 30100 Spain; 5 Department of Human Anatomy and Psychobiology, Faculty of Psychology, University of Murcia, Murcia, 30100 Spain; 6 Department of Anatomy, Histology and Neuroscience, School of Medicine, Autonomous University of Madrid, Madrid, Spain; 7 Department of Anatomy and Cell Biology, University of Illinois Chicago - College of Medicine, Chicago, IL 60612 USA

**Keywords:** Forebrain, Prosomeres, Hindbrain, Diencephalon, VTA, SN

## Abstract

The Mongolian Gerbil (*Meriones unguiculatus*) diverged from rats/mice around 45 million years ago and developed adaptations to extreme temperatures and water scarcity. Another feature of the Mongolian Gerbils is their social monogamy similar to that of prairie voles. These observations suggest that there are potential differences in the Mongolian Gerbil brain that are distinct from that of rats and mice. The goal of the present study is to establish the extent to which the neuromeric organization of the brain is conserved in the Mongolian Gerbil and to gain insights on how evolutionary expansion and diversification of brain regions occur across species. Our data shows that the multineuromeric origin of tyrosine hydroxylase-positive processes in the Mongolian Gerbil is similar to that in mice and rats, spanning from the diencephalon, midbrain, and the rostral hindbrain. There are also observable anatomical differences. However, most of the components characteristic of these neuromeres are identifiable in the Mongolian gerbil, closely mirroring those found in mice and rats. Together, these findings suggest that the conserved neuromeric organization likely stems from a restricted genetic toolset that began in the Muridae family 45 million years ago, and that a profound reorganization of the fundamental structural plan delineating the neuromeric segmentation is not required for the emergence of diverse functionality among species of phylogenetically related families. Future studies are needed to establish how the genetic programs within each neuromeric unit are influenced by environmental factors that ultimately impact the size of the neuromeric derivatives and their functional connectivity.

## Introduction

**Fig. 1:**
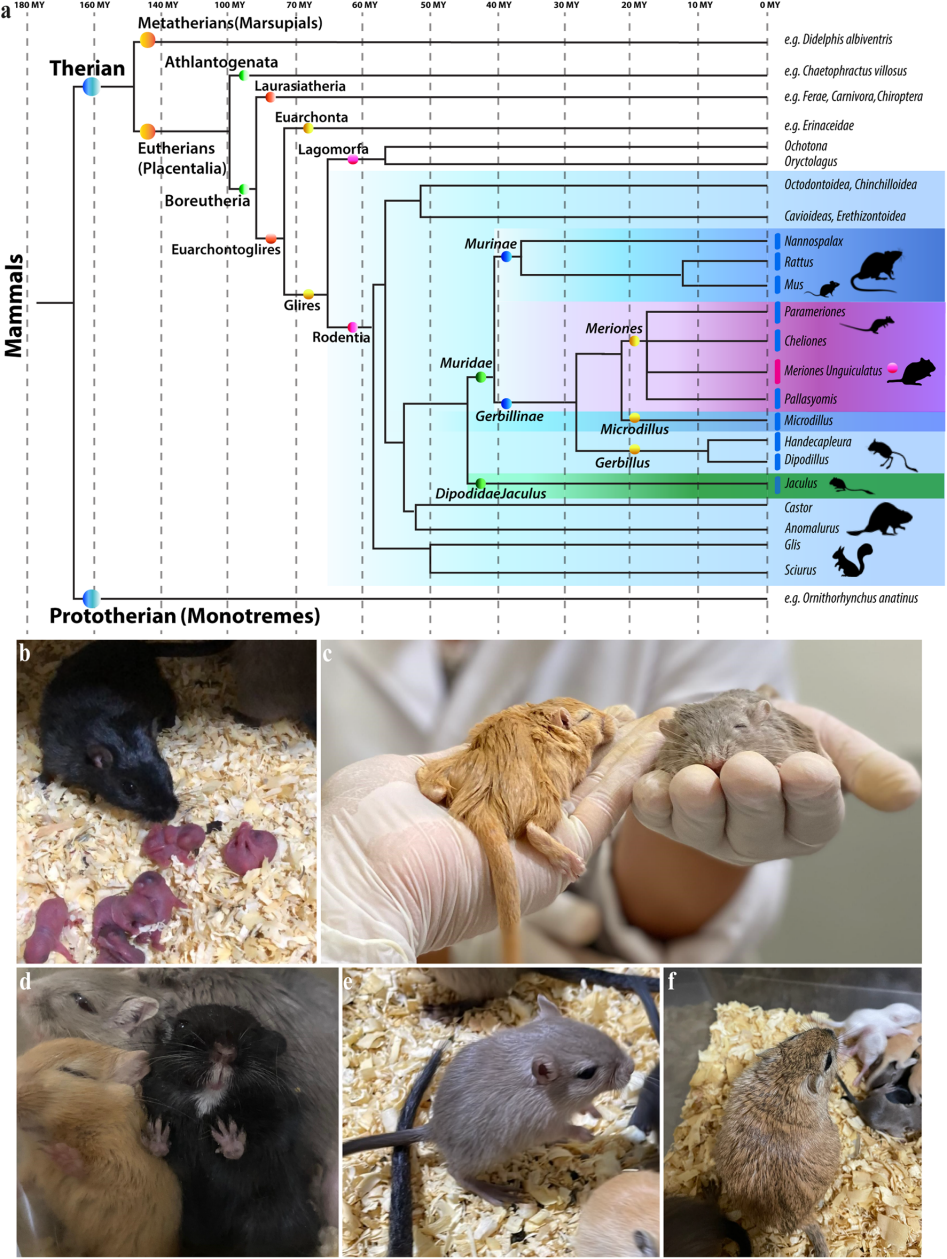
**a** Simplified phylogenetic tree of mammals illustrating the evolutionary relationships among the major subfamilies Muridae, including Murinae (e.g., rats and mice) and Gerbillinae, the subfamily of *Meriones unguiculatus*. While Rodentia and Lagomorpha shared a common ancestor approximately 75 million years ago. However, the Muridae family emerged around 45 million years ago, subsequently diverging into the Murinae and Gerbillinae subfamilies approximately 40 million years ago (Voloch et al. 2013; Delsuc et al. 2016; Halliday et al. 2019; Prevosti et al. 2021; Foley et al. 2023). **b**–**f** The Mongolian gerbil (*Meriones unguiculatus*) shares a mouse-like appearance but with some notable differences. They are covered in dense, soft fur that typically ranges from a sandy to a reddish-brown on their back, fading to a creamy white on their belly. A prominent feature is their long, tufted tail, often matching the length of their head and body combined and ending in a brush-like tuft of darker fur. This tail is crucial for balance, particularly when the gerbil stands upright. Beyond their appearance, *Meriones unguiculatus* exhibits remarkable adaptations to arid environments. These include highly efficient water conservation, evidenced by their ability to produce concentrated urine and dry feces, and their skill in acquiring most of their water from food. Their powerful hind legs and natural burrowing behavior further enable them to navigate and thrive in dry, desert-like conditions

The Mongolian Gerbil (*Meriones unguiculatus*, (Thomas 1908), also referred to as the “gerbil”, is a small, omnivorous rodent belonging to the Gerbillinae subfamily (Fig. 1a). A common ancestor of the subfamilies Gerbillinae and Murinae (which includes rats and mice) likely existed approximately 45 million years ago within the family Muridae (Fig. 1a) (Voloch et al. 2013; Delsuc et al. 2016; Halliday et al. 2019; Prevosti et al. 2021; Foley et al. 2023). The gerbil has a mouse-like appearance, characterized by dense fur, a long-tufted tail and adaptations for arid environments (Fig. 1b–f). Native to the sandy, semi-desert regions of Mongolia and northern China, this species thrives in extreme environments featured by temperature fluctuations and limited water availability (Gromov 2021). Beyond its ecological niche, the Mongolian Gerbil has been used in biomedical and neuroscience research, particularly in the field of auditory processing and cerebrovascular function (Ryan 1976; Ohl et al. 1999; Traystman 2003). A unique feature of the Mongolian Gerbil is the incomplete Circle of Willis, which allows for controlled induction of cerebral infarction, making it a preferred model for stroke and ischemia studies (Traystman 2003). In addition, the Mongolian gerbil is a versatile model for developmental neuroscience research and aging-related studies (Dawirs et al. 1997; Rupniak et al. 2003; Tziridis et al. 2015; Deliano et al. 2020). Mongolian gerbils are also known for social monogamy, a rare reproductive strategy among rodents, similar to that of prairie voles (*Microtus ochrogaster*). This trait suggests shared neurodevelopmental and behavioral mechanisms underlying pair-bonding and social behavior (McGraw and Young 2010; Gromov 2021). Anatomical and connectivity studies, but also interspecies comparisons to determine homologues derivatives of gerbil with other vertebrates will be improved using standardized references like that offered by the prosomeric framework.

**Fig. 2:**
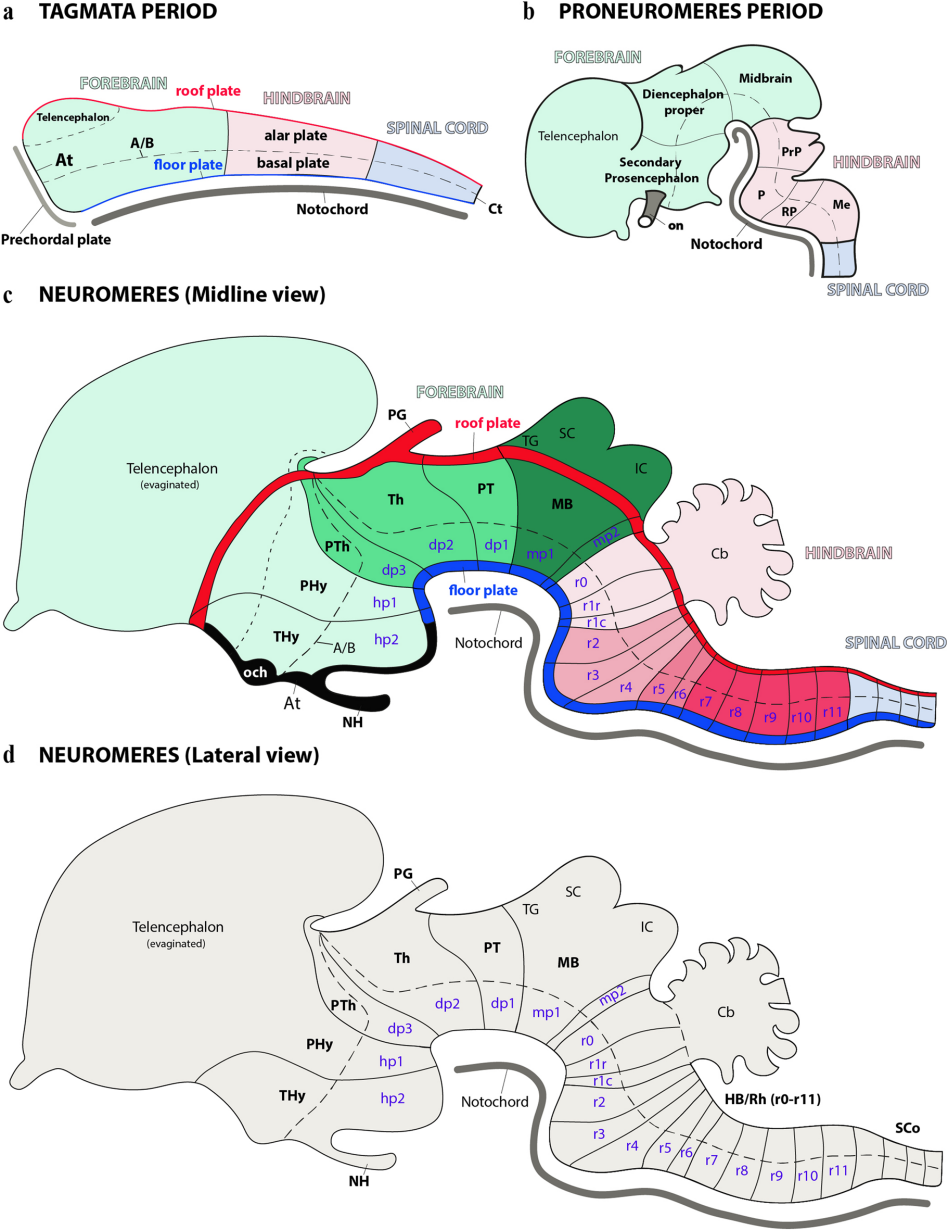
A series of schematic drawings which visualize the sequential anterior-posterior (AP) neural regionalization during development, interpreted through the lens of the prosomeric framework. Key early, intermediate, and late stages of this process, which ultimately leads to complete neuromeric segmentation, are highlighted (See below). **a** Upon neural tube closure, the neural tube establishes its fundamental organization, dividing into three distinct anteroposterior compartments: the forebrain (at the rostral end), the hindbrain, and the spinal cord (extending caudally). Concurrently, these partitions, often termed ‘tagmata’, are accompanied by the dorsoventral patterning of the neural tube, which establishes four fundamental domains: the roof, alar, basal, and floor plates. The acroterminal (At) and caudal terminal (Ct) domains identify where the alar and basal plates converge at the neural tube’s rostral and caudal ends, respectively. The prechordal plate, situated adjacent to the neural tube’s most rostral end, significantly influences early neural tube regionalization. Meanwhile, the notochord spans the entire rostrocaudal axis and provides crucial signals for dorsoventral regionalization, the main impact of which is observed in the floor and basal plates. In this scheme, pale green denotes the forebrain, pale pink represents the hindbrain, and pale blue signifies the spinal cord. **b** During the proneuromeric stage, the forebrain regionalizes into three main anterior posterior parts: the secondary prosencephalon (rostral), the diencephalon proper, and the midbrain (caudal). Simultaneously, the hindbrain organizes into four distinct regions from rostral to caudal: the prepontine (PrP), pontine (P), retropontine (RP), and medullary territories (Me). The telencephalon develops by evaginating from the alar plate of the secondary prosencephalon, while the superior colliculus (SC) and inferior colliculus (IC) originate from the alar plate of the midbrain. To maintain visual consistency, forebrain derivatives are represented in shades of green, hindbrain derivatives in pink, and spinal cord-related derivatives in pale blue. **c** As development progresses into the neuromeric period, the fundamental building blocks of the brain, the neuromeres, are fully established. A lateral schematic view, incorporating the midline, reveals that each of these individual neuromeres is characterized by its own specialized roof, alar, and basal floor plates. The secondary prosencephalon gives rise to two hypothalamo-prosencephalic prosomeres (hp1 and hp2). Moving caudally, the diencephalon proper forms three distinct diencephalic prosomeres (dp1-dp3). The midbrain then differentiates into two prosomeres (mp1 and mp2). Further back, the hindbrain exhibits a complex organization of thirteen segments known as rhombomeres (r0-r11). The pineal gland (PG) emerges as a derivative of the diencephalic roof plate and the optic chiasma (och) and neurohypophysis (NH) are derived from the At domain. **d** Lateral schematic view illustrating the neuromeric partitioning along the rostrocaudal axis of the brain, emphasizing the distinct alar and basal territories within each neuromere. A/B: alar-basal boundary; Cb: Cerebellum; IC: Inferior colliculus; OB: Olfactory bulb; PHy: Peduncular hypothalamus; MB: Midbrain; PG: Pineal gland; PT: Pretectum; PTh: Prethalamus; SC: Superior colliculus; SCo: Spinal cord; TG: Tectal gray; THy: Terminal hypothalamus; Th: Thalamus. Refer to the list for full anatomical abbreviations

According to the prosomeric framework, at early stages of development, the central nervous system can be divided into three regions known as primary tagmata: the forebrain (prosencephalon), hindbrain (rhombencephalon), and spinal cord (Fig. 2a) (Albuixech-Crespo et al. 2017; Ferran and Puelles 2018; Ferran et al. 2022). The forebrain can be further subdivided into three proneuromeres along the rostral to caudal axis: secondary prosencephalon, diencephalon proper and midbrain (Fig. 2b, c). Similarly, the hindbrain regionalizes into four proneuromeres, identified rostrocaudally as the prepontine (PrP), pontine (P), retropontine (RP), and medullary (Me) regions (Fig. 2b, c). Similarly, each neural tube region is further subdivided along the anteroposterior axis. This process creates fundamental transversal units, termed neuromeres, which are structurally defined by their unique roof, alar, basal and floor plates (Fig. 2c, d). These neuromeres, also known as prosomeres in the forebrain and rhombomeres in the hindbrain, are considered developmental modules that sculpt the brain’s structural and functional diversity (Fig. 2c, d) (Puelles and Rubenstein 1993, 2003, 2015; Puelles et al. 2012a, b, c; Ferran et al. 2015c; Watson et al. 2017; Puelles 2019; Puelles and Hidalgo-Sánchez 2023). Thus, the prosomeric framework provides a unique working model for understanding the development of neural circuit processes by integrating molecular patterning with evolutionary conserved anatomical boundaries across vertebrates (Puelles and Rubenstein 2003, 2015; Puelles 2009, 2019; Puelles and Ferran 2012; Ferran 2017). Current observations suggest that each neuromere acts as a foundational unit, governed by distinct genetic programs that dictate its specialization and connectivity. This modular organization is conserved throughout vertebrate evolution, underscoring its fundamental role in brain development and diversification (Puelles 2013, 2018, 2019; Puelles and Rubenstein 2015; Ferran 2017).

**Fig. 3:**
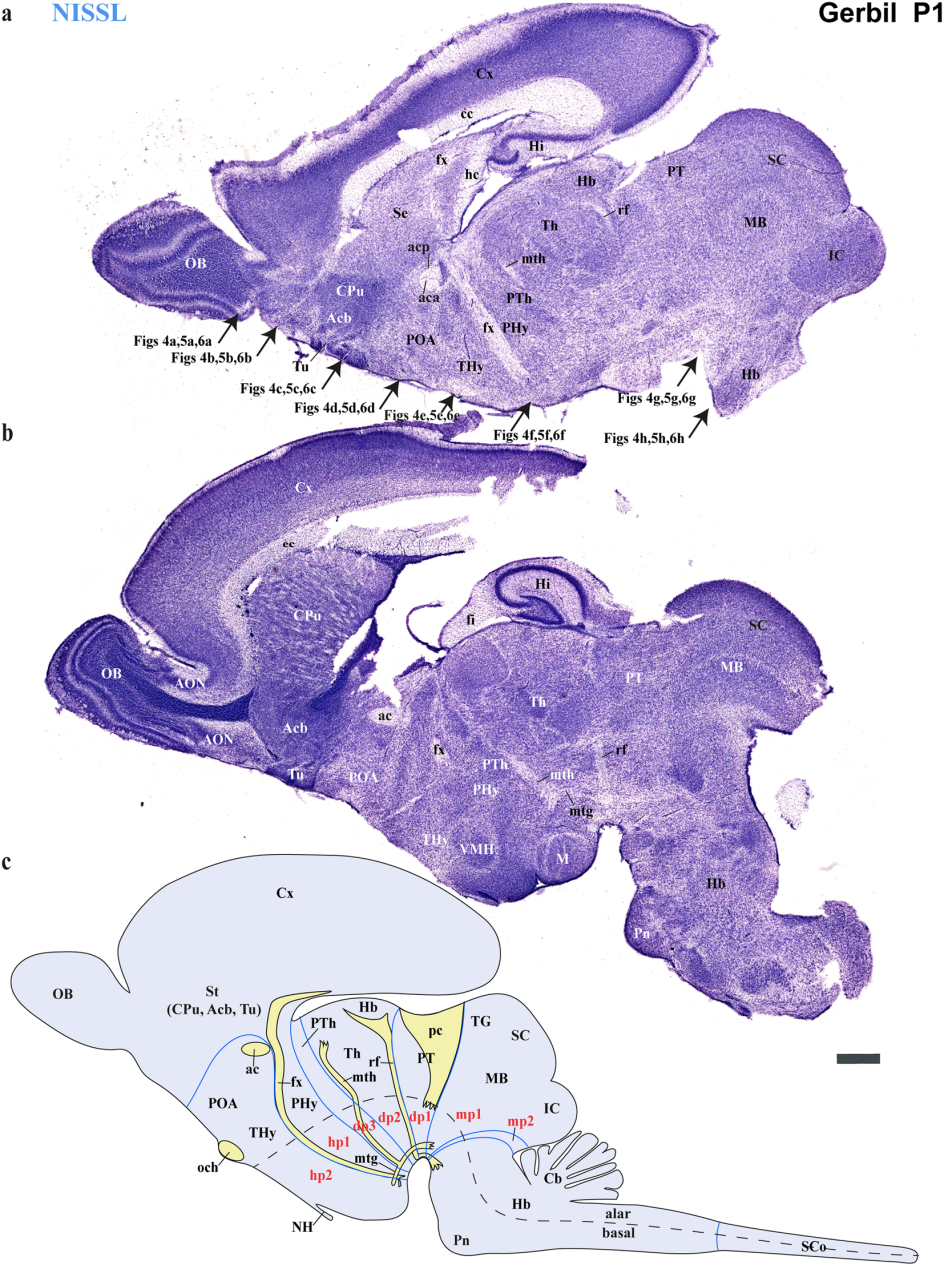
Selected consecutive Nissl-stained sagittal sections through the secondary prosencephalon, diencephalon proper, midbrain, and hindbrain of the Mongolian gerbil at postnatal day1 (P1), compared with a schematic view of the neuromeric subdivisions. **a** The secondary prosencephalon encompasses telencephalic derivatives, specifically as part of the most dorsal alar plate of the hypothalamic peduncular (PHy) and terminal (THy) neuromeres. Structures like the Cortex (Cx), olfactory bulb (OB), hippocampus (Hi), and striatal derivatives (caudate/putamen-CPu-, accumbens-Acb-, and olfactory tubercle-Tu-) are observable within the PHy. In contrast, the subpallial preoptic area (POA) belongs to the terminal neuromere (THy). The telencephalon contains derivatives of the commissural plate, such as the corpus callosum (cc), hippocampal commissure (hc), and anterior commissure (ac). Additionally, the alar and basal plates of the hypothalamus in the PHy and THy neuromeres are observed, with the rostral border of the fornix (fx) indicating the boundary between these neuromeres. The diencephalic neuromeres are discernible by locating the mammillothalamic tract in the alar plate of dp2 (diencephalic prosomere 2). This allows for the identification of the prethalamus (dp3) rostrally and the thalamus caudally (dp2). The retroflex tract (rf) is observed detaching from the habenular region (Hb) and extending towards the basal plate of dp2. The caudal border of this tract limits with the pretectal region (dp1). Note that the Hb belongs to dp2. The diencephalic region transitions into the midbrain, where both the superior and inferior colliculi are clearly discernible. Additionally, the rostral hindbrain is identified in this section. Arrows indicate the plane of section for the corresponding images in Figs. 4 and 5, and 6. **b** A more lateral sagittal section from the same specimen offers enhanced visualization of many previously indicated neuroanatomical features. Notably, the caudate/putamen is now visible in relation to the external capsule. The fimbria of the hippocampus is also detected in close proximity to the choroideal tela. The hypothalamus in the PHy and THy neuromeres is more visible, allowing for the identification of specific THy derivatives like the ventromedial (VMH) and mammillary body (M). Furthermore, the pontine nuclei are clearly discernible at the hindbrain level. **c** Schematic lateral view of the Mongolian brain showing its neuromeric distribution and emphasizing the key tracts used to define its boundaries. Fornix (fx) fibers can be seen emerging from the hippocampus; with part of the tract passing behind the anterior commissure to reach the mammillary body. The mammillothalamic (mth) and mammillotegmental (mtg) tracts arise from the mammillary body (M), extending to the thalamic anterior nuclei and hindbrain tegmentum, respectively. The retroflex tract originates from the habenular region, reaching the interpeduncular nucleus. The posterior commissure (pc) signal in its caudal border the limit between diencephalon and midbrain. Refer to the list for full anatomical abbreviations. A scale bar of 500 μm is applied to all images

**Fig. 4:**
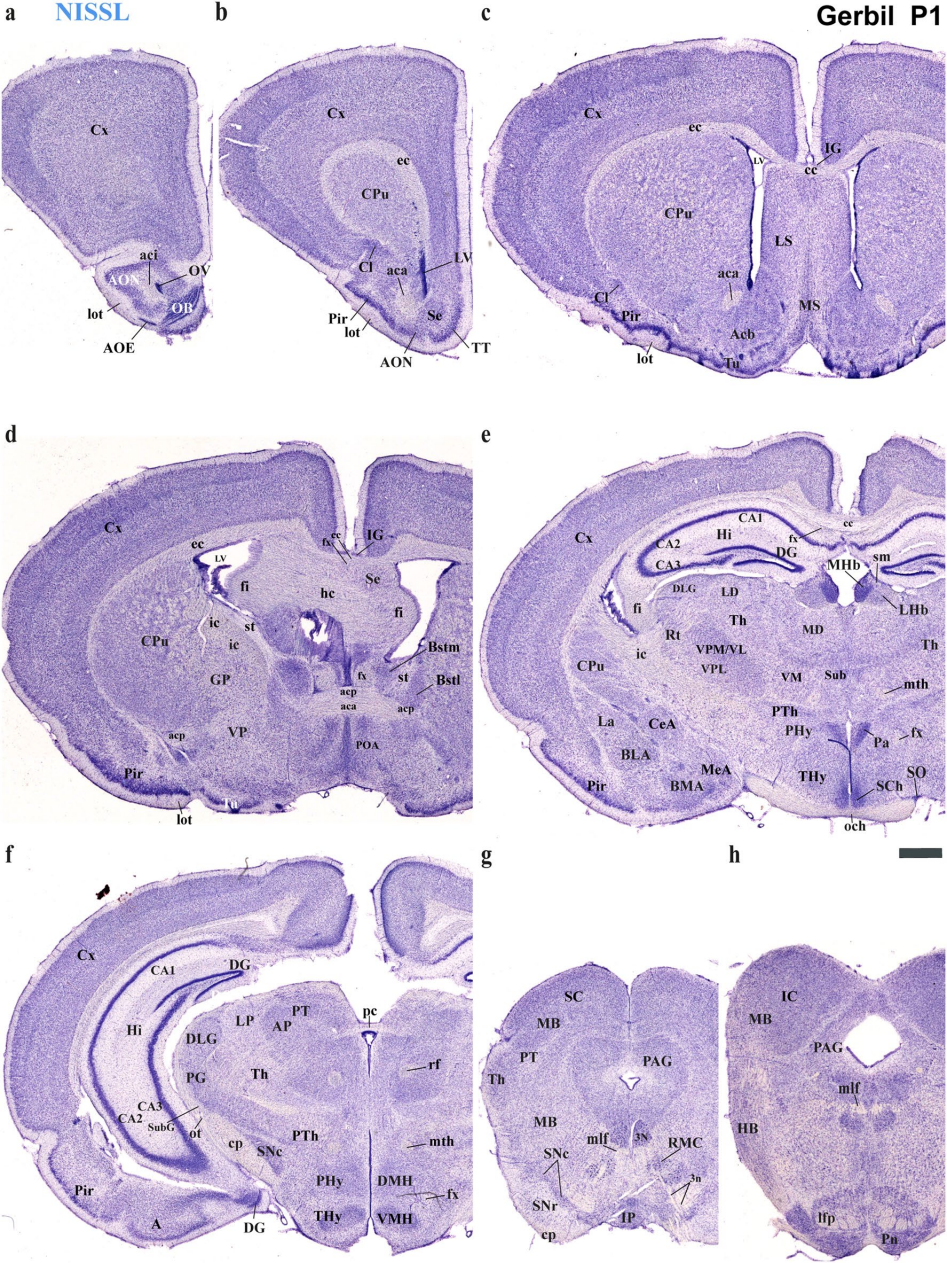
Selected rostrocaudal sequence of consecutive Nissl-stained classical cross-sections of postnatal day Mongolian gerbil 1 (P1) (section plane indicated for each image in Fig. 3a). **a**–**d** Selected sections showing telencephalic derivatives like cortex (Cx), caudate-putamen (CPu), claustrum (Cl), piriform cortex (Pir), lateral septum (LS), medial septum (MS), accumbens (Acb), pallidal derivatives (GP) and Olfactory bulb (OB) mostly belonging to the PHy, but some of them to the THy (e.g. POA). These images also highlight the derivatives of the commissural plate (anterior commissure -ac-, corpus callosum -cc- and hippocampal commissure -hc-) and the fornix (fx) tract. **e**, **f** Selected sections mainly show hypothalamic (PHy, THy) and diencephalic (dp1-dp3) derivatives with references to the fornix (fx), mamillothalamic (mth), retroflex (rf) and posterior commissure (pc) tracts that help to localize interneuromeric boundaries. Within the terminal hypothalamic neuromere (THy), the suprachiasmatic (SCh) and ventromedian hypothalamic (VMH) nuclei are observed. In the PHy, the paraventricular (Pa) and dorsomedian (DMH) hypothalamic nuclei are visible. As part of the diencephalon proper, prethalamic (dp3; e.g. PG, Rt), thalamic (dp2; e.g. VPL, MD, VM), habenular (dp2; MHb, LHb) and pretectal (dp1; AP) derivatives can be observed. These sections also reveal important telencephalic derivatives, primarily from the PHy neuromere (Cx, Hi and amygdala-e.g. BLA, BMA, CeA, La, MeA-). Note that the mth tract in (**e**) is located on the alar plate of dp2, indicating the boundary between dp2 and dp3. However, in (**f**) it is located on the basal plate of dp3. At its caudal border, the posterior commissure (pc) signals the limit between the diencephalon and the midbrain. **g**, **h** Selected consecutive images illustrating midbrain structures, including the superior colliculus (SC), periaqueductal gray (PAG), and inferior colliculus (IC), as well as hindbrain derivatives such as the interpeduncular nucleus (IP) and pontine nuclei (Pn). Refer to the list for full anatomical abbreviations. A scale bar of 500 μm is applied to all images

**Fig. 5:**
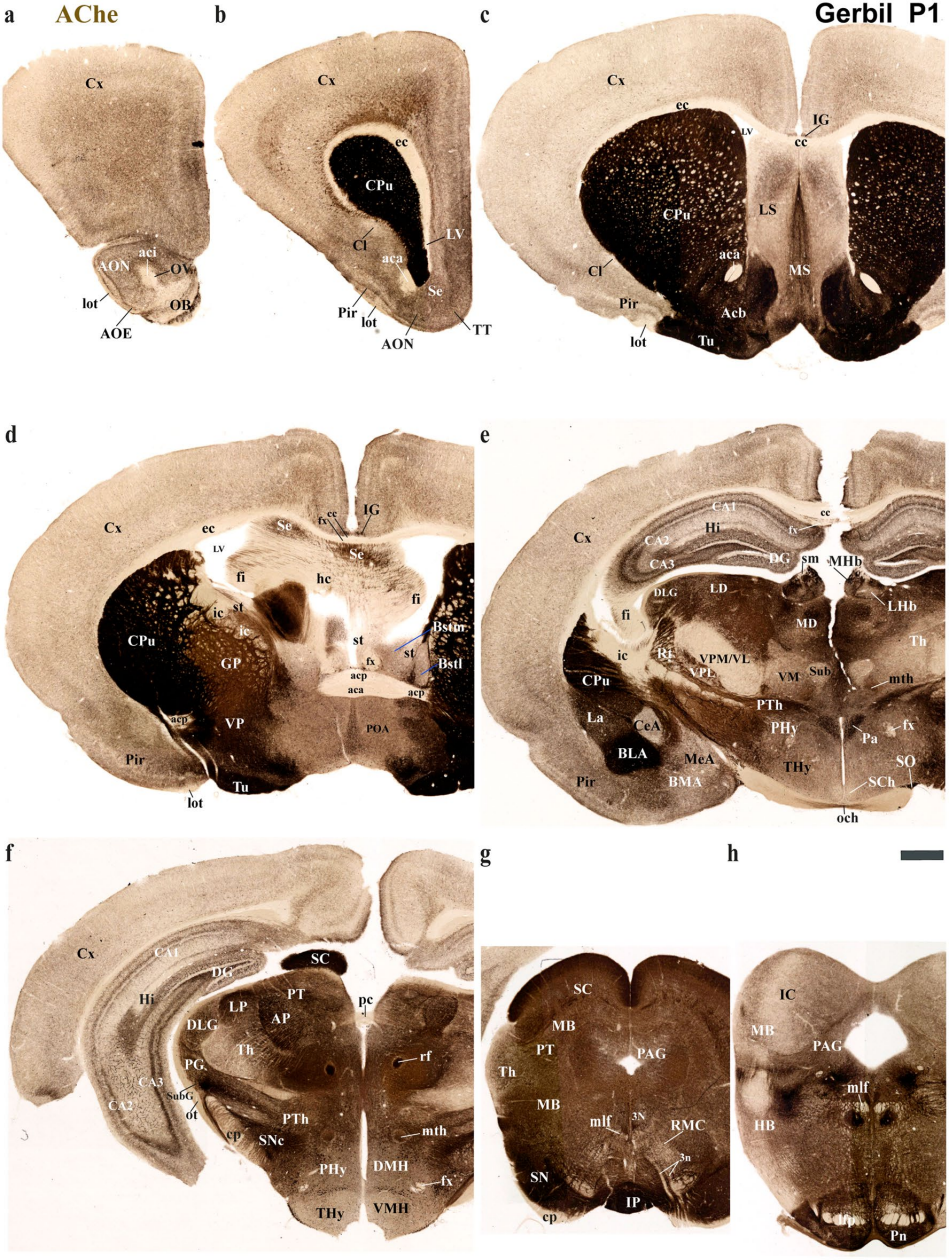
Selected rostrocaudal sequence of consecutive classical cross-sections of acetylcholinesterase (AChE) activity, from the same postnatal day 1 (P1) Mongolian gerbil described in Figs. 4 and 6 (section plane indicated for each image in Fig. 3a). **a**–**d** Selected sections showing telencephalic derivatives mostly belonging to the PHy (e.g., cortex (Cx), caudate-putamen (CPu), claustrum (Cl), piriform cortex (Pir), lateral septum (LS), medial septum (MS), accumbens (Acb), pallidal derivatives (GP), Olfactory bulb (OB)) but also to the THy (e.g. POA) neuromere. AChE staining effectively highlights the striatal and pallidal derivatives. The striatum, organized as a radial domain, includes the caudate-putamen (CPu) nucleus nearest the ventricle, the accumbens (Acb) nucleus, and finally, the most superficial structure, the olfactory tubercle (Tu), towards the pial surface. Differential staining patterns were observed within the lateral (LS) and medial (MS) septal nuclei. Additionally, AChE activity was evident in the indusium griseum (IG) (compare with Nissl and Gallyas stains in Figs. 4d and 6d). The contrast created by this reaction highlights the position of the inner capsule (ic), stria terminalis (st) tract, corpus callosum (cc), anterior commissure (ac), hippocampal commissure (hc), fornix and fimbria hippocampus (fi). Note that the st tract is between the bed nuclei of stria terminales medial and lateral (**d**). **e**, **f** Selected sections showing hypothalamic (THy, PHy) and diencephalic derivatives (dp1-dp3), referencing key tracts like the fornix (fx), mammillothalamic (mth), retroflex (rf), and posterior commissure (pc) to aid in localizing interneuromeric boundaries. The signal is practically absent in the suprachiasmatic (SCh) and ventromedial nuclei (VMH) of the THy. In contrast, the paraventricular nucleus (Pa) of the PHy exhibits a strong signal. Within the diencephalon, AChE activity is notably absent in certain thalamic nuclei (e.g. VPM/VL) and very low in others (e.g., VMC, Sub). Conversely, the intense signal in the habenular region distinctly highlights the stria medullaris (sm) tract. AChE reaction in these sections effectively visualizes key telencephalic derivatives, largely originating from the PHy. These include the Cortex (Cx), Hippocampus (Hi), and components of the amygdala, such as the basolateral (BLA), basomedial (BMA) central (CeA) lateral (La), and medial (MeA) nuclei. Notably, a differential AChE staining is observed across the hippocampal regions (DG, CA1, CA2, CA3) and within the amygdala nuclei (The signal is practically absent in the central amygdala (CeA) and decreased in the medial amygdala (MeA), but it’s higher in the lateral (La) and basolateral (BLA) amygdala). A positive signal is also observed in the retroflex (rf) and mamillothalamic (mth) tracts. **g**, **h** Selected images showing midbrain (e.g. SC, PAG, IC) and hindbrain (e.g. IP, Po) derivatives. The strongest signal is observed in the superficial layers of the superior colliculus (SC), substance nigra (SN), interpeduncular nucleus (IP) and pontine nuclei (Pn). Refer to the list for full anatomical abbreviations. A scale bar of 500 μm is applied to all images

**Fig. 6:**
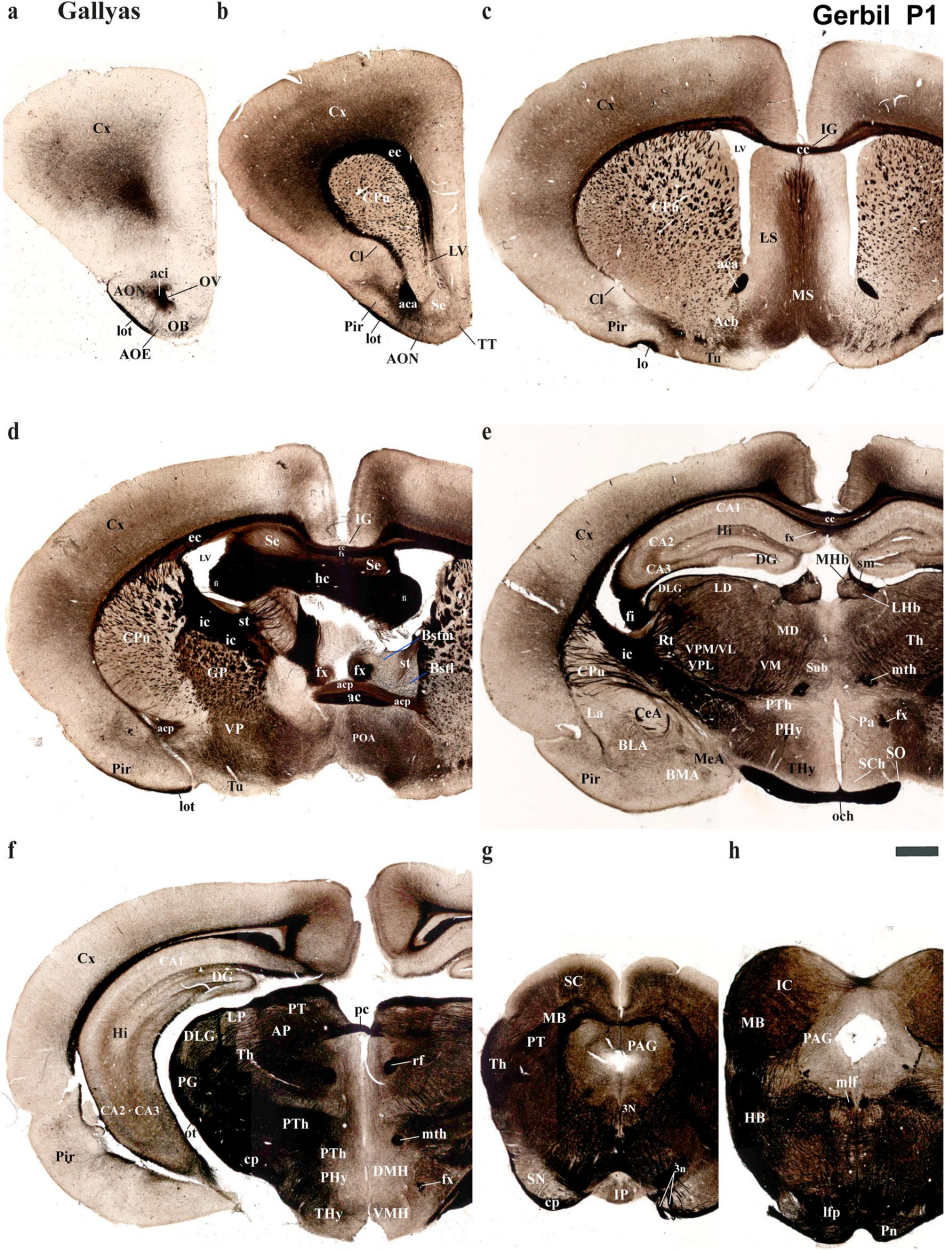
Selected rostrocaudal sequence of consecutive classical cross-sections of Gallyas staining, from the same postnatal day 1 (P1) Mongolian gerbil described in Figs. 4 and 5 (section plane indicated for each image in Fig. 3a). **a**–**d** Selected sections showing telencephalic derivatives mostly belonging to the PHy (e.g., cortex (Cx), caudate-putamen (CPu), claustrum (Cl), piriform cortex (Pir), lateral septum (LS), medial septum (MS), accumbens (Acb), pallidal derivatives (GP), Olfactory bulb (OB)) but also to the THy (e.g. POA) neuromere. Gallyas staining effectively visualizes the major tracts within the telencephalic region, including both its evaginated and unevaginated components. The external capsule (ec) is clearly seen enveloping the striatal derivatives. This tract, along with the midline corpus callosum, is distinct from the underlying fornix (**c**, **d**). Gallyas staining highlights main tracts coursing through the telencephalic region (evaginated and inpar). The external capsule (ec) is observed covering striatal derivatives. This tract followed by corpus callosum (cc) in the midline is observed but also differentiated from the body of fornix under it (**c**, **d**). Both the anterior (aca) and posterior (acp) parts of the anterior commissure are visible. The anterior part (aca) is seen in its intrabulbar portion (aci, see image **a**) and as it courses towards the midline (**b**–**d**). The posterior part (acp), which primarily connects amygdalar derivatives, is also observed as a distinct portion at the midline (**d**). The inner capsule (ic), hippocampal commissure (hc) and stria terminalis(st) are also observed. Note that the st tract is between the bed nuclei of stria terminales medial and lateral (**d**). **e**–**f** Selected sections showing hypothalamic (THy, PHy) and diencephalic derivatives (dp1-dp3), referencing key tracts like the fornix (fx), mammillothalamic (mth), retroflex (rf), and posterior commissure (pc) to aid in localizing interneuromeric boundaries. The optic chiasm (och), inner capsule (ic), cerebral peduncle (cp.), fimbria hippocampus (fi), stria medularis (sm) are also highlighted. **g**, **h** Selected images showing midbrain (e.g. SC, PAG, IC) and hindbrain (e.g. IP, Po) derivatives. Note the strong signal in the cerebral peduncle and longitudinal fasciculus pons (lfp). Refer to the list for full anatomical abbreviations. A 500 μm scale bar applies for all images

Using the prosomeric framework, we recently showed that TH-positive neurons within the substantia nigra (SN) and ventral tegmental area (VTA) originate from multiple neuromeres across both rodents and primates (Ferran et al. 2025). The rationale for using the prosomeric framework for understanding the development of distinct neural circuits is the fact that the neuromeric/segmental organization is conserved across vertebrates (Puelles and Rubenstein 1993, 2003, 2015; Puelles and Ferran 2012), which is a key fundamental principle to gain insights on how evolutionary expansion and diversification of brain regions occur across species (Puelles and Rubenstein 1993, 2003, 2015; Puelles and Ferran 2012; Albuixech-Crespo et al. 2017; Puelles 2018). Detailed studies on closely related rodent species, similar to those conducted between chickens and quails, are necessary to determine if their anatomical boundaries and connectivity are representative of their evolutionary clade (Merchan et al. 2011). Regardless of the observed ecological niche and behavioral specificities of the Mongolian Gerbils, we expected a high degree of neuromeric conservation when compared to mice and rats (members of the *Murinae* family). Thus, the goal of the present study is to employ the same strategy to track TH-positive processes as a proxy to reveal whether a similar pattern of brain development exists in the Mongolian gerbil. Specifically, we will first identify distinct neuromeric units with their interneuromeric boundaries known to be conserved across species primates (Ferran et al. 2025) and assess the extent to which such anatomical landmarks apply to the Mongolian gerbil brain at two distinct developmental stages: postnatal day (P) 1 and P55. We will then delineate the multi-neuromeric origin of TH-positive neurons within the SN and VTA and compare the trajectories of TH-positive processes projecting to the striatum (SN-striatum) and to the limbic system (VTA-accumbens and VTA-prefrontal cortex). Using classical histological techniques such as Nissl staining, Acetylcholinesterase (AChE) histochemistry, and Gallyas silver myelin staining, we were able to identify complementary cytoarchitectural, chemoarchitectural, and myeloarchitectural features of neuromeric partitions in the Mongolian gerbil brain (Figs. 3, 4, 5 and 6).

## Materials and methods

All animal procedures received approval from the relevant institutional ethics committees. Specifically, the Advisory Committee for the Care and Use of Experimental Animals of the Faculty of Veterinary Sciences of the University of La Pampa approved the use of Mongolian gerbils. Animal housing complied with the FORCED guidelines (Garrigos et al. 2021).

### Rodents

Mongolian gerbils at postnatal day 1 (P1) and postnatal day 55 (P55) were acquired from the animal facilities at the University of La Pampa. Upon arrival, all animals were weighed and then housed in standard cages (50 × 35 × 35 cm). These cages were equipped with 2–3 cm of cork bedding. Environmental conditions in the housing facility were consistently maintained at 22–25 °C with 45–60% humidity. Animals were provided with *ad libitum* access to standard chow (ENVIGO 2014S) and filtered water.

### Rodent brain tissue processing

Nine Mongolian gerbil brains at P1 and 6 brains at P55 (male and female) were collected and processed using established protocols (Ferran et al. 2015a, b). Briefly, animals were deeply anesthetized with an intraperitoneal injection of pentobarbital (100 mg/kg), followed by an intramuscular injection of ketamine (10 mg/kg). After a transcardial perfusion with saline, the animals were perfused with 4% paraformaldehyde (PFA) in 0.1 M phosphate buffer (PB, pH 7.4). The brains were then removed and post-fixed in PFA at 4 °C for 24 h. For free-floating immunohistochemistry sections, brains were embedded in 4% agarose (Pronadisa #8008, Spain) and sectioned sagittal or transversal at 100 μm using a Leica Vibratome system. Sections were then mounted on SuperFrost Plus slides for immunohistochemical analysis protocols (Ferran et al. 2015a, b). A subset of brains was chosen for the examination of cytoarchitecture, myeloarchitecture, and chemoarchitecture. This involved utilizing Nissl staining, Gallyas silver myelin staining (Gallyas 1979), and acetylcholinesterase histochemistry (AChE) (Cavada et al. 1995). Prior to sectioning, these brains underwent cryoprotection in 30% phosphate-buffered sucrose. They were then cut into 50 μm coronal or sagittal sections using a freezing microtome.

### Nissl

Tissue sections were mounted on gelatin-coated slides and dried for 10 days. The sections were then rinsed in a 0.1 M, pH 7.4 phosphate buffer (PB). For defatting, they were submerged in a 1:1 solution of chloroform and 100% ethanol for 3 h. Next, the sections were rehydrated by passing them through a series of descending ethanol solutions (100%, 95%, 70% for 20 min each) and then in distilled water for 5 min. The sections were stained with 0.1% cresyl violet (pH 3.8) for 3 min, followed by a 5-minute rinse in distilled water. Dehydration and differentiation were performed by moving the sections through a series of ascending ethanol solutions (70%, 95%, 100% for 20 min each). Then, the sections were cleared in xylenes. All the steps were carried on at room temperature. Finally, sections were cover slipped with mounting media (Entellan, Sigma-Aldrich).

### Gallyas

Sections were first rinsed in distilled water and then incubated for 30 min in a solution containing two parts pyridine and one part glacial acetic acid. After this, they were again rinsed in distilled water. Next, sections were placed in the impregnation solution for 30 min in the dark. This solution was prepared by dissolving 0.1 g each of ammonium nitrate and silver nitrate in 100 ml of distilled water, and its pH was adjusted to 7.5 with 0.1 M sodium hydroxide. Following impregnation, sections were rinsed in 0.5% acetic acid and transferred to the developing solution. The developing solution’s incubation time was determined by microscopic observation to achieve the desired staining intensity. The developing solution was prepared in a specific order: 150 ml of Solution A (25 g sodium carbonate in 500 ml distilled water) was mixed with 75 ml of Solution B (1 g ammonium nitrate, 1 g silver nitrate, and 5 g silicontungstic acid in 500 ml distilled water), and then 75 ml of Solution C (73.25 ml of Solution B and 1.75 ml of 4% paraformaldehyde) was added. After development, sections were rinsed in 1% acetic acid, followed by a rinse in distilled water. They were then stabilized for 30 min in the dark using a 5% sodium thiosulfate solution prepared in distilled water. All steps were conducted at room temperature. Finally, sections were rinsed in distilled water, mounted in a 0.1 M, pH 7.4 phosphate buffer (PB), and cover-slipped using a standard mounting medium (Entellan, Sigma-Aldrich) (Gallyas 1979).

### Acetylcholinesterase

Sections were rinsed in distilled water and then incubated overnight at 4 °C in the AChE solution. This solution was prepared with 0.2 mM ethopropazine hydrochloride, 4 mM acetylthiocholine iodide, 10 mM glycine, 2 mM cupric sulfate pentahydrate, and 50 mM sodium acetate in distilled water, with the pH adjusted to 5.0 with acetic acid. Following incubation, sections were rinsed in distilled water and then immersed in an 8 mM sodium sulfide solution for 2–5 min at room temperature in the dark. The pH of this solution was adjusted to 7.8 with 3 N hydrochloric acid. A final rinse in distilled water was performed. The reaction was intensified by incubating the sections for 30 min in the dark with a 0.5% silver nitrate solution prepared in distilled water. After this step, the sections were differentiated and stabilized by incubation for 30 min in the dark with a 5% sodium thiosulfate solution prepared in distilled water. All preceding steps were carried out at room temperature unless otherwise specified. Finally, sections were rinsed, mounted in a 0.1 M, pH 7.4 phosphate buffer (PB), and cover-slipped with a mounting medium (Entellan, Sigma-Aldrich) (Cavada et al. 1995).

### Immunohistochemistry

We performed immunohistochemistry based on a previously published protocol (Ferran et al. 2015a, b). Briefly, we inactivated endogenous peroxidases in tissue sections using 0.3% hydrogen peroxide. Sections were then incubated for 48 h at 4 °C with the primary antibody, rabbit anti-TH (NB300-109, Novusbio, Bio-Techne R&D Systems, Spain; 1:200), followed by a 24-hour incubation with biotinylated secondary antibodies (goat anti-rabbit IgG (H + L), Vector Laboratories, BA-1000-1.5; 1:200). Next, sections were exposed to a streptavidin-peroxidase complex (Vectastain-ABC kit, Vector Laboratories, United States; PK4000) for 2 h at room temperature. Peroxidase activity was visualized using 0.03% 3,3’-diaminobenzidine (DAB, Sigma, St. Louis, MO, United States) with 0.003% hydrogen peroxide. The specificity of the TH primary antibody in rodents has been previously confirmed (Bilbao et al. 2022; Ferran et al. 2025), We observed no residual immunostaining in experiments conducted without the primary antibody.

Imaging

For image acquisition, tissue sections were digitally scanned with an Aperio Technologies ScanScope CS system (magnification x20). Subsequent image adjustments, including modifications to size, contrast, brightness, and focus, were carried out in Adobe Photoshop 2025. The final figures were then meticulously assembled using Adobe Illustrator 2025 (Adobe Systems).

## Results

The primary focus of the present study was to identify the neuromeric components and their interneuromeric boundaries in the Mongolian gerbil brain at postnatal day (P) 1 and P55. The analyses began with the forebrain, which includes the secondary prosencephalon, diencephalon proper, and the midbrain. Similar sequential analyses were applied to the hindbrain that includes the prepontine, pontine, retropontine and medullary regions.

### Secondary prosencephalon of the Mongolian gerbil

**Fig. 7:**
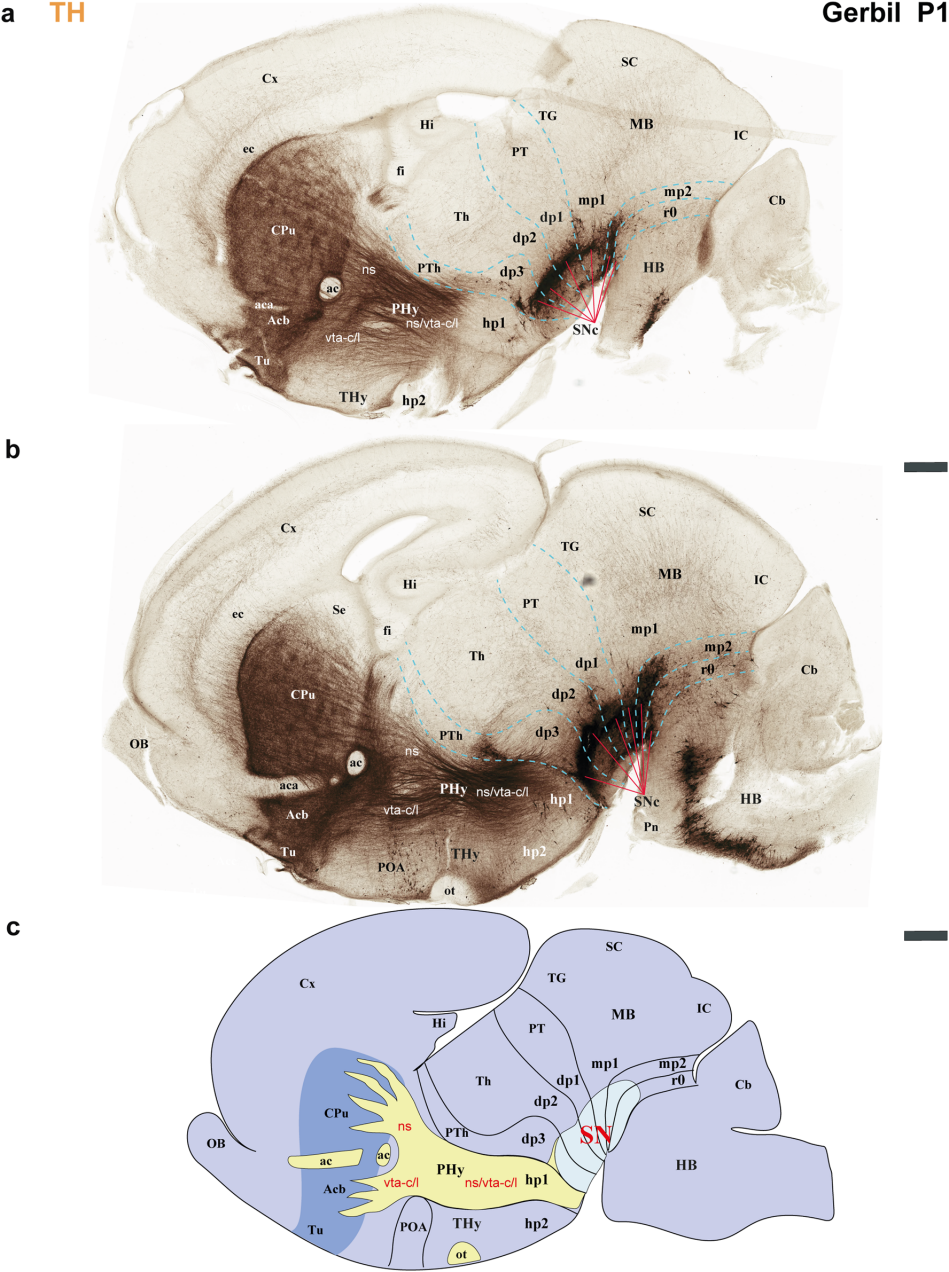
**a**–**c** A lateral-to-medial series of sagittal sections from postnatal day 1 (P1) Mongolian gerbils, processed for tyrosine hydroxylase (TH) immunohistochemistry, reveals the neuromeric distribution of substantia nigra (SN). This pattern further indicates the location of the nigro-striatal (ns) tract, the pathway connecting the substantia nigra (SN) to the caudate-putamen (striatum). Furthermore, the fibers that connect the VTA to the nucleus accumbens through the VTA-limbic tract (vta-l), and to the prefrontal cortex via the VTA-cortical tract (vta-c) follow this same path. **a** This section reveals the substantia nigra pars compacta (SNc) extending across the diencephalic (dp1-dp3), midbrain (mp1, mp2), and rostral hindbrain (r0) neuromeres. Observe the nigro-striatal (ns), VTA-limbic (vta-l), and VTA-cortical (vta-c) tracts as they pass through the peduncular hypothalamus (PHy). Although these tracts run close to the pial surface, they are covered by the cerebral peduncle (cp.). The signal is intense in the striatal derivatives (CPu, Acb, and Tu). These tracts, as they course through the PHy, indicate the rostral boundary with the terminal hypothalamus (THy) and the caudal boundary with the prethalamus (dp3). **b** This consecutive more medial section shows aspects similar to the previous one. Additionally, the course of the anterior commissure is visible, and the nigro-striatal (ns), VTA-limbic (vta-l), and VTA-cortical (vta-c) tracts are present across nearly the entire basal plate of the PHy. **c** A schematic lateral view of the brain of Mongolian gerbil, based on the prosomeric framework, illustrates the multineuromeric distribution of the substantia nigra (SN). Additionally, the diagram illustrates the dorsoventral trajectory of the nigro-striatal (ns), VTA-limbic (vta-l), and VTA-cortical (vta-c) tracts as they pass through the peduncular hypothalamus (PHy). Refer to the list for full anatomical abbreviations. A 500 μm scale bar applies for each image

**Fig. 8:**
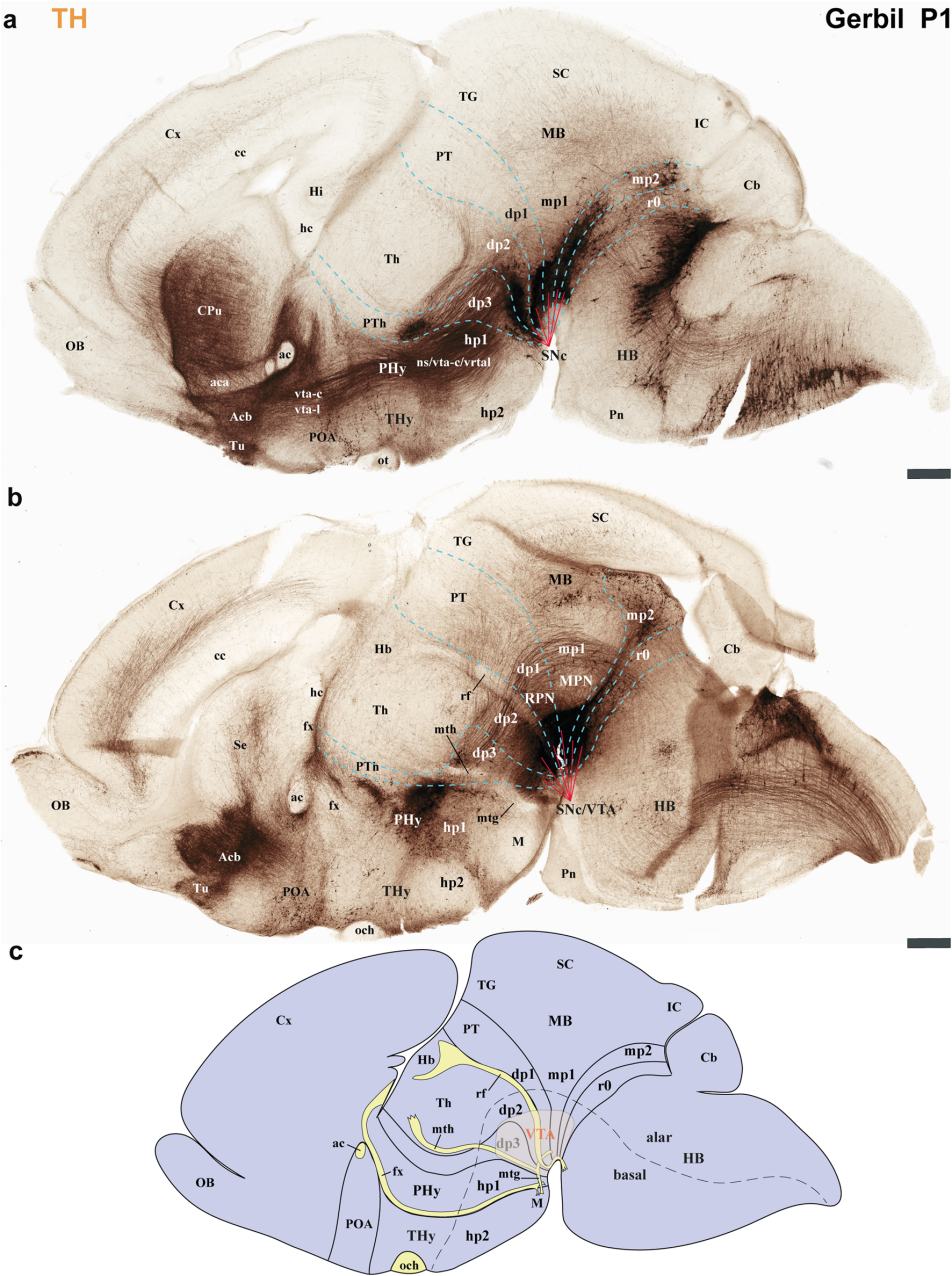
**a**–**c** This figure extends the lateral-to-medial sagittal section series (initiated in Fig. 7) of postnatal day 1 (P1) Mongolian gerbils. Processed with tyrosine hydroxylase (TH) immunohistochemistry, these sections further elucidate the neuromeric distribution of the substantia nigra (SN) and the ventral tegmental area (VTA). This pattern further indicates the location of the fibers that connect the VTA to the nucleus accumbens through the VTA-limbic tract (vta-l), and to the prefrontal cortex via the VTA-cortical tract (vta-c). **a** Similar to Fig. 7, the substantia nigra pars compacta (SNc) display a multi-neuromeric distribution across dp1-dp3, mp1-mp2, and r0. The VTA-limbic (vta-l) and VTA-cortical (vta-c) tracts appear to reach and pass through the accumbens nucleus, respectively. However, their dorsoventral course through the peduncular hypothalamus (PHy) importantly marks the border between the PHy and the terminal hypothalamus (THy), as well as between the PHy and dp3 (prethalamus). The nucleus accumbens (Acb) is positioned superficially to the anterior commissure (ac) but deep to the olfactory tubercle (Tu). **b** This consecutive, more medial section reveals the mammillothalamic (mth) and retroflex (rf) tracts. The rostral border of the mammillothalamic tract delineates the boundary between the thalamus (dp2) and the prethalamus (dp3) at the alar plate level. Conversely, the caudal border of the retroflex tract indicates the boundary between the thalamus (dp2) and the pretectum (dp1). Both tracts are crucial for recognizing the multineuromeric location of the substantia nigra (SN) and ventral tegmental area (VTA) components. **c** A schematic lateral view of the brain of Mongolian gerbil, based on the prosomeric framework, illustrates the multineuromeric distribution of the ventral tegmental area (VTA). The schema also illustrates the course of the fornix (fx), mammillothalamic (mth), and retroflex (rf) tracts, serving as useful landmarks for identifying several interneuromeric boundaries. Refer to the list for full anatomical abbreviations. A 500 μm scale bar applies for each image

**Fig. 9:**
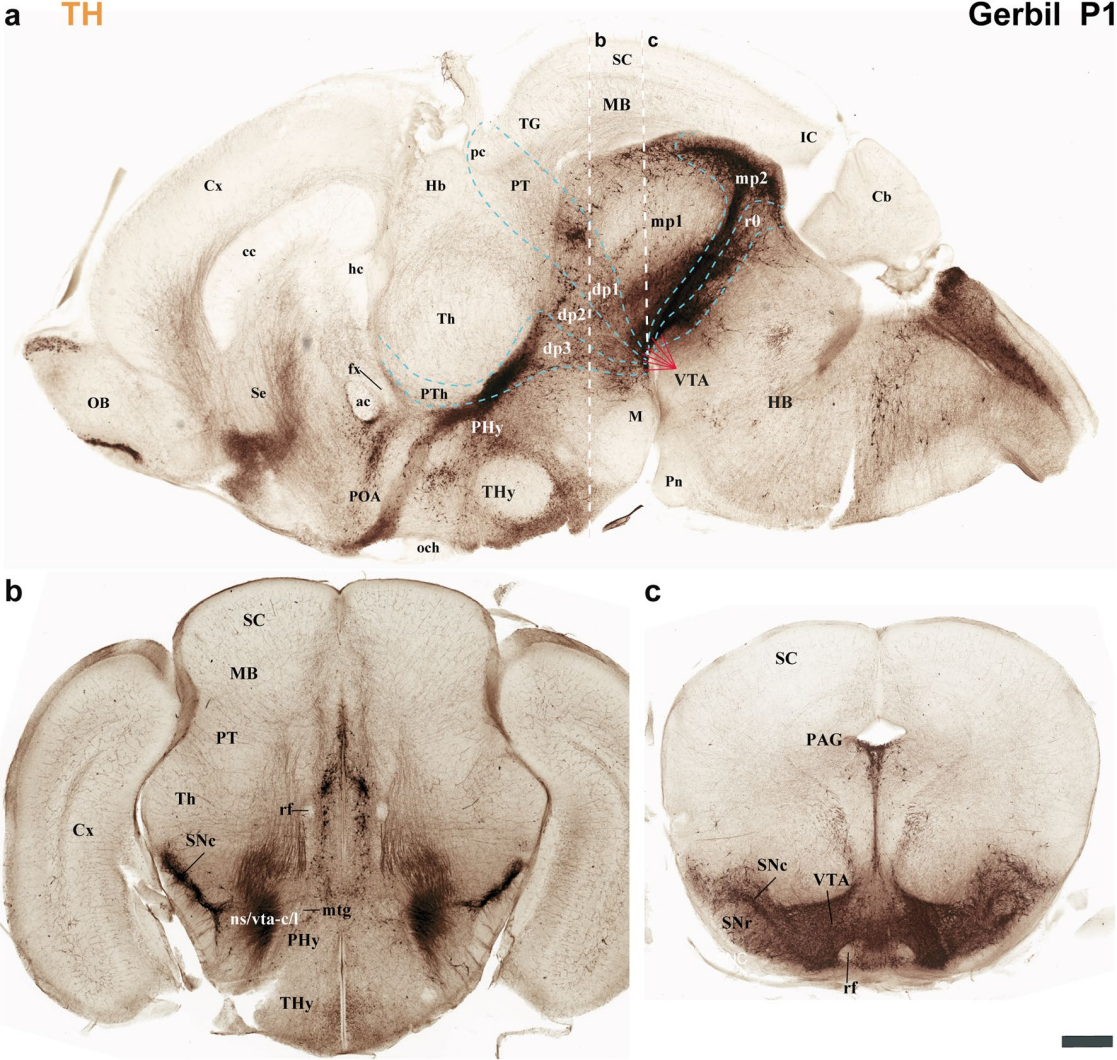
This figure extends the lateral-to-medial sagittal section series (initiated in Fig. 7) of postnatal day 1 (P1) Mongolian gerbils. Processed with tyrosine hydroxylase (TH) immunohistochemistry, these sections further elucidate the neuromeric distribution of the ventral tegmental area (VTA). **a** Similar to Fig. 8, the ventral tegmental area (VTA) displays a multi-neuromeric distribution across dp1-dp3, mp1-mp2, and r0. **b** As indicated in image (**a**), this horizontal section, parallel to the rostrocaudal axis in the forebrain, from a postnatal day 1 (P1) Mongolian gerbil passes through the secondary prosencephalon (PHy, THy), diencephalon proper (dp1-dp3), and midbrain (mp1). The substantia nigra pars compacta (SNc) is detected in close proximity to the thalamus (dp2) and the prethalamus (dp3). **c** This selected consecutive section, from the same animal as in (**b**), shows tyrosine hydroxylase (TH) expression in the VTA and SN (see image ‘a’ for the plane of section). Refer to the list for full anatomical abbreviations. A 500 μm scale bar applies for all images

**Fig. 10:**
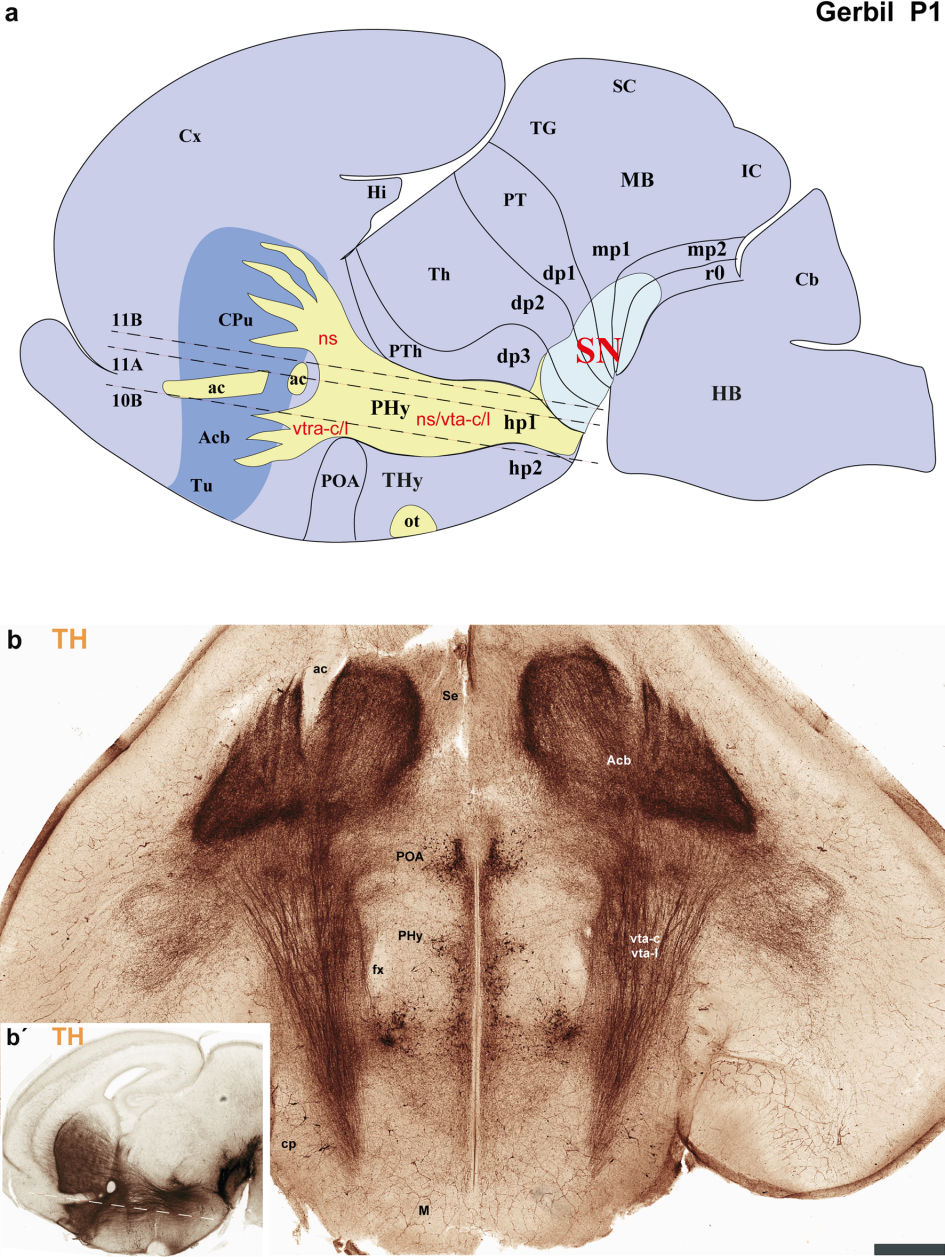
**a** A schematic lateral view of the brain of Mongolian gerbil, based on the prosomeric framework, which illustrates the multineuromeric distribution of the substantia nigra (SN). Additionally, the diagram illustrates the ventrodorsal trajectory of the nigro-striatal (ns), VTA-limbic (vta-l), and VTA-cortical (vta-c) tracts as they pass through the peduncular hypothalamus (PHy). The plane of section of image (**b**) and Fig. 11 is indicated. **b** A transversal section of the peduncular hypothalamus (PHy) from a postnatal day 1 (P1) Mongolian gerbil processed for tyrosine hydroxylase (TH) immunohistochemistry. This section plane is perpendicular to the anteroposterior axis of the neural tube, indicating a true transversal cut through the PHy prosomere. **b** The most rostral selected transversal sections to the peduncular hypothalamus (PHy) illustrate the probable disposition of the VTA-limbic (vta-l) and VTA-cortical (vta-c) tracts as they reach the accumbens nucleus and prefrontal cortex, respectively. Note the orientation of this section plane in the sagittal view shown in (**b**’). Refer to the list for full anatomical abbreviations. A 500 μm scale bar applies for each image

**Fig. 11:**
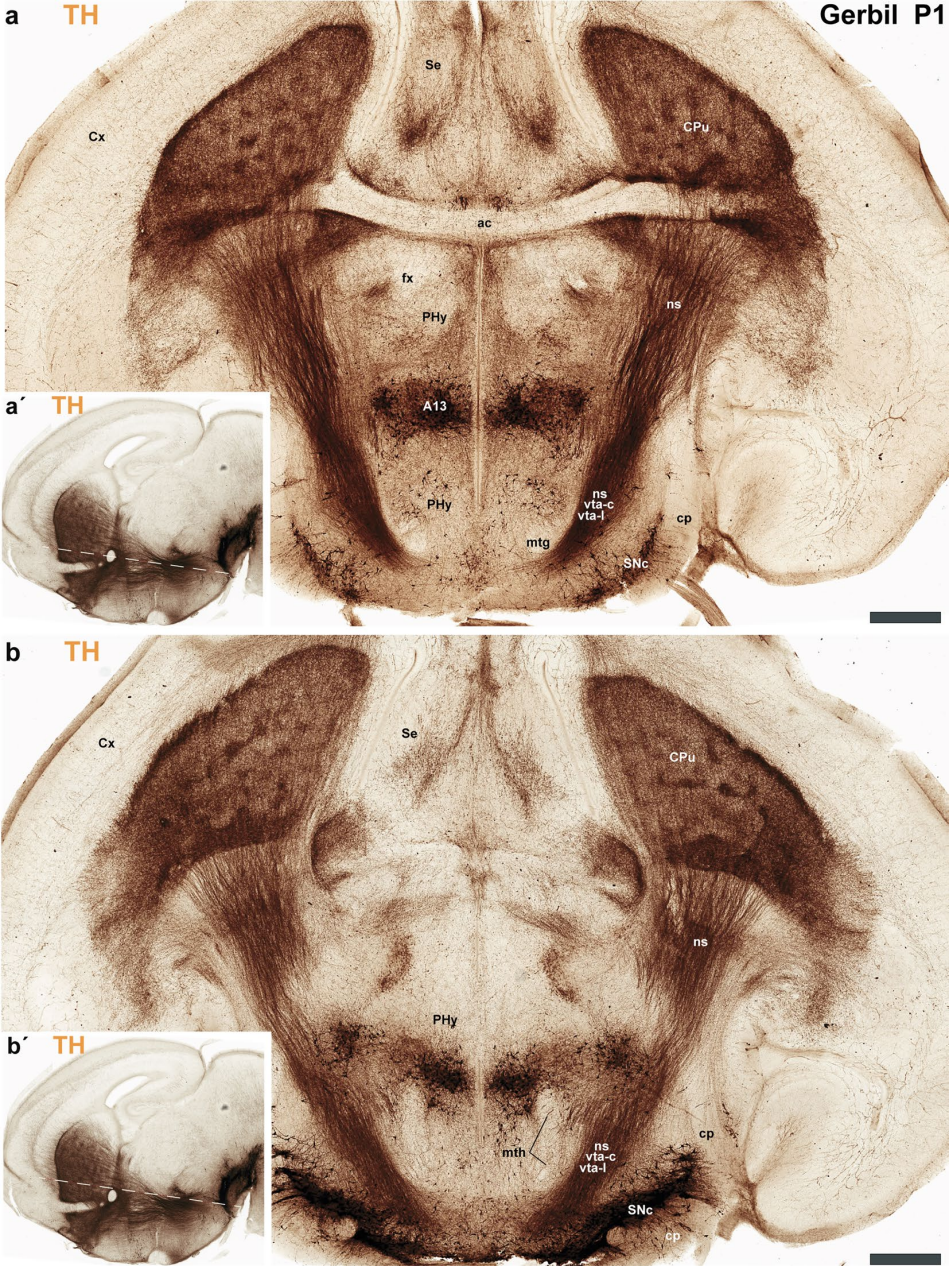
**a**–**b**´ Processed for tyrosine hydroxylase (TH) immunohistochemistry, these selected transversal sections from the peduncular hypothalamus (PHy) of a postnatal day 1 (P1) Mongolian gerbil clearly show the ventrodorsal trajectory of the nigro-striatal (ns), VTA-limbic (vta-l), and VTA-cortical (vta-c) tracts as they pass through this region. **a** This consecutive section from the same animal as Fig. 10 shows the nigro-striatal (ns), VTA-limbic (vta-l), and VTA-cortical (vta-c) tracts following a ventrodorsal disposition. However, close to the anterior commissure, axons arriving at the caudate-putamen (CPu) are most probably expected to belong to the nigro-striatal (ns) tract. Note that these tracts are located deep to the cerebral peduncle (cp.) during their traverse through the PHy. Additionally, observe the presence of the substantia nigra pars compacta (SNc), situated in the basal plate of the diencephalic prosomere 3 (dp3). The plane of section is shown in (**a**´) but also in Fig. 10a. **b** The most caudal consecutive transversal section selected shows the nigro-striatal (ns) tract reaching the caudate-putamen nucleus. Additionally, observe the substantia nigra located in dp3. The plane of section is shown in (**b**´) but also in Fig. 10a. Refer to the list for full anatomical abbreviations. A 500 μm scale bar applies for each image

**Fig. 12:**
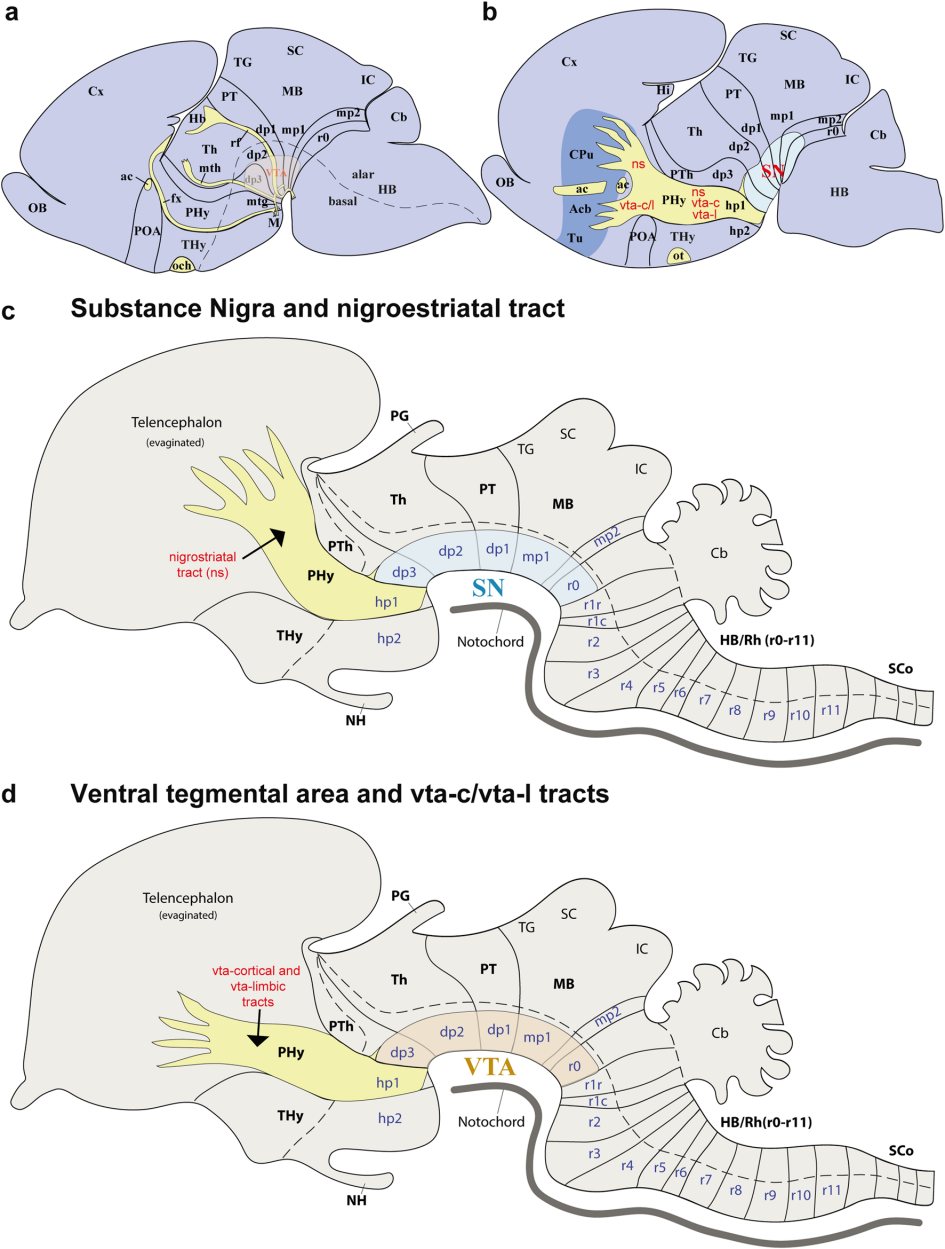
**a**–**d** Schematics lateral views illustrating the prosomeric framework of the Mongolian gerbil, interpreting the anatomical positions of the substantia nigra (SN), ventral tegmental area (VTA), and their respective tracts. **a**, **b** Schematic lateral views of the Mongolian gerbil CNS illustrate its neuromeric subdivisions in both lateral and more medial sections. This interpretation reveals the locations of the nigro-striatal (ns), VTA-limbic (vta-l), and VTA-cortical (vta-c) tracts, which are visible in lateral sections. Furthermore, it depicts the substantia nigra (SN) spanning multiple neuromeric segments (**a**). In more medial sections, the multineuromeric location of the VTA in the Mongolian gerbil becomes apparent. Furthermore, the visibility of crucial tracts like the fornix (fx), mammillothalamic (mth), and retroflex (rf) tracts aids to identify interneuromeric boundaries. **c** This general schematic represents the prosomeric framework, highlighting the multineuromeric location of the substantia nigra (SN) and nigrostriatal (ns) tract. **d** This general schematic represents the prosomeric framework, highlighting the multineuromeric location of the ventral tegmental area (VTA) and its associated VTA-limbic (vta-l) and VTA-cortical (vta-c) tracts. Refer to the list for full anatomical abbreviations

**Fig. 13:**
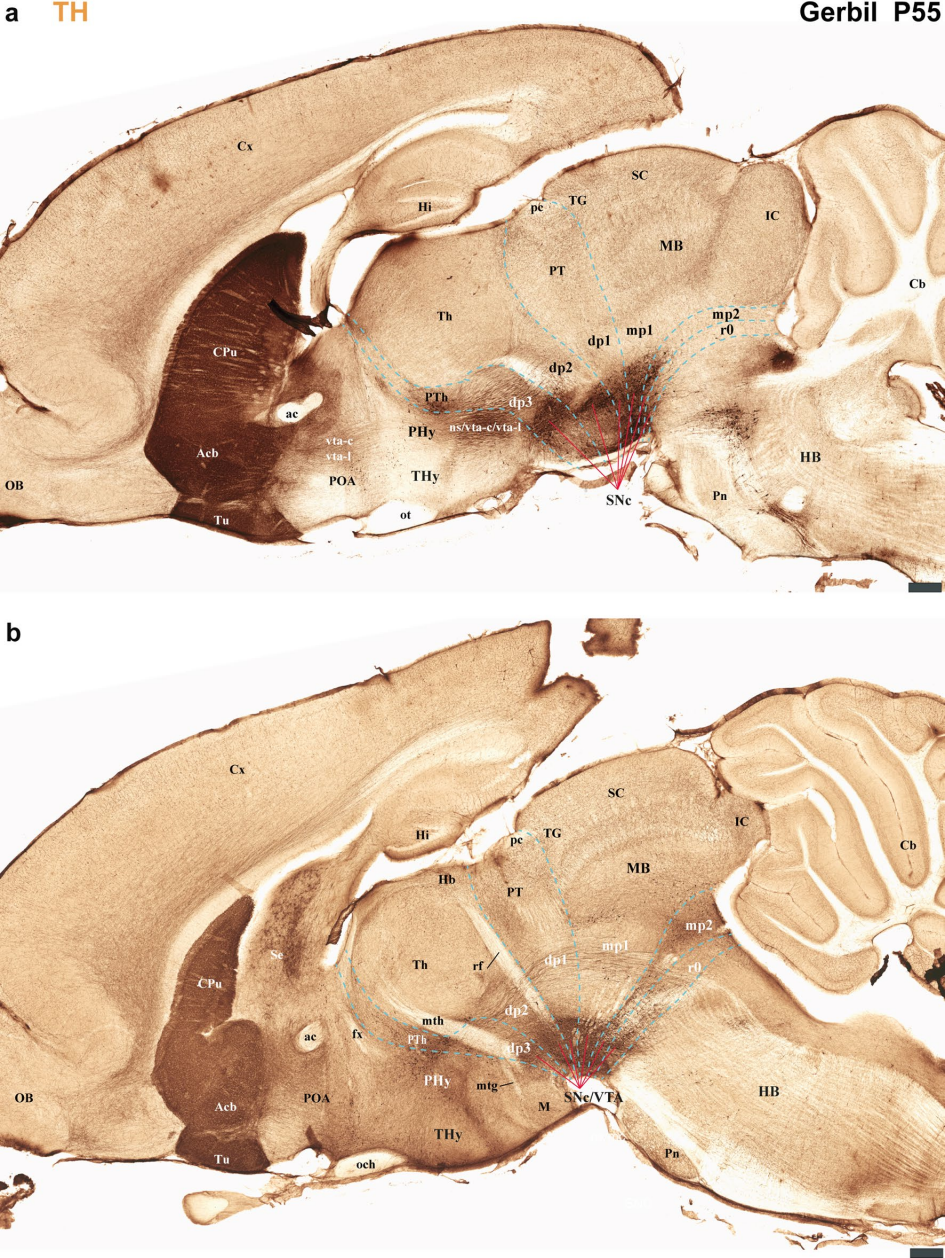
**a**, **b** Selected sections from a lateral-to-medial series of sagittal sections obtained from a postnatal day 55 (P55) Mongolian gerbil, processed for tyrosine hydroxylase (TH) immunohistochemistry. **a** This lateral section displays TH immunoreaction within the striatum and substantia nigra (SN). It also shows the initial segments of the nigro-striatal (ns), VTA-limbic (vta-l), and VTA-cortical (vta-c) tracts in the basal plate of the peduncular hypothalamus (PHy). The SN itself is observed distributed across the basal plate of the diencephalon proper (dp1-dp3), midbrain (mp1, mp2), and rostral hindbrain (r0). **b** A more medial section reveals the fornix (fx), mammillothalamic (mth), retroflex tract (rf), and posterior commissure (pc), all of which aid in localizing interneuromeric boundaries. Notably, the retroflex tract (rf), as it reaches the basal plate of dp2, divides the SN and VTA into a rostral part (with dp2 and dp3 components) and a caudal part (with dp1, midbrain [mp1, mp2], and r0 derivatives). Refer to the list for full anatomical abbreviations. A 500 μm scale bar applies for each image

The secondary prosencephalon is the most rostral region of the neural tube. This proneuromere contains two distinct neuromeres: the peduncular hypothalamo-telencephalic prosomere (hp1), also known as the peduncular hypothalamus (PHy), and the terminal hypothalamo-telencephalic prosomere (hp2), or terminal hypothalamus (THy). Each of these neuromeres has its own roof, alar, basal and floor plates (Fig. 2c, d) (Puelles et al. 2012b; Puelles and Rubenstein 2015; Ferran et al. 2015c). These neuromeres and their boundaries can be identified by distinct anatomical landmarks, which are observable at both postnatal day 1 (P1) and P55 of gerbils. The dorsal alar plate of the PHy (hp1) is a crucial area, containing both pallial components (e.g., the cortex) and subpallial components (e.g., the striatum). These components are directly continuous with the alar hypothalamus (e.g. paraventricular nucleus) (Figs. 2c and d, 3a–c, 4a–c, 5a-c, 6a–c, 7a–c, 8a–c and 13) (Ferran et al. 2015c; Puelles et al. 2016; Puelles and Rubenstein 2015). A key anatomical landmark is the fornix tract, which distinguishes the hp1-hp2 boundary in the alar and basal plates of the hypothalamus. This tract (white matter bundle) originates from the hippocampus and projects to the mammillary body. Its trajectory within the PHy, caudal to the anterior commissure and directly next to the rostral edge of the hp1 prosomere’s alar and basal plates, make it a precise anatomical reference. Therefore, the rostral border of the fornix tract marks the transition point between the hp1 and hp2 prosomeres (Figs. 3a–c, 4d–f, 5d–f, 6d–f, 8b, c, 9a and 13b) (Bilbao et al. 2022; Ferran et al. 2025). The hp1 and hp2 boundaries can be further identified by the rostral edge of distinct trajectories of tyrosine hydroxylase (TH)-positive tracts along PHy (hp1). These pathways originate from the ventral tegmental area (VTA) and substantia nigra (SN), projecting to specific forebrain regions: the caudate-putamen of the striatum (via the nigrostriatal tract, ns), the prefrontal cortex (via the VTA-cortical tract, vta-c), and the nucleus accumbens (via the VTA-limbic tract, vta-l) (Figs. 7a–c, 8a, c and 12) (Bilbao et al. 2022; Ferran et al. 2025). TH staining allows for meticulous tracking of these axons in the Mongolian gerbil (Figs. 7, 8, 9, 10, 11, 12 and 13). These tracts initially follow a caudo-rostral trajectory, extending rostrally into the peduncular hypothalamus (hp1) (Figs. 7a–c, 8a, 12, 13a) (Ferran et al. 2025). Upon reaching the basal plate of the hp1 prosomere, these axons make a sharp 90-degree turn. They then navigate through both the basal and alar plates of the peduncular hypothalamus to innervate their striatal, cortical, and limbic targets (Figs. 7a–c, 8a, 10, 11, 12, 13a). As the trajectory of these tracts follows through the hypothalamus, they are found beneath the cerebral peduncle (cp.), though they maintain a relatively superficial position near its pial surface (Figs. 10, 11). The caudal boundary of the TH-positive tracts that travel through hp1 also helps to delineate the hp1-dp3 border (hypothalamic-diencephalic) (Figs. 7a, c, 8a and 12) (Bilbao et al. 2022; Ferran et al. 2025).

The Mongolian gerbil’s terminal hypothalamus (THy or hp2), the most rostral neuromere of the neural tube, is characterized by the presence of the preoptic area (POA). This area, a subpallial telencephalic component, is located in the dorsal most alar plate, near the roof plate and is also continuous with the alar hypothalamus. Additionally, prominent derivatives such as the suprachiasmatic, arcuate, ventromedial, and mammillary body nuclei, are found within the hypothalamic alar and basal plates of hp2 of Mongolian gerbil (Figs. 3a–c, 4d–f, 5d–f, 6d–f, 7b, c, 8a–c, 9a, b and 13a, b) (Bardet et al. 2010; Puelles et al. 2012b, 2016; Puelles and Rubenstein 2015; Bilbao et al. 2022).

### Diencephalon proper of the Mongolian gerbil

The diencephalon proper is composed of three diencephalic prosomeres (dp1–dp3); pretectum (dp1), thalamus (dp2), and prethalamus (dp3) (Fig. 2c, d) (Puelles et al. 2012c; Puelles and Rubenstein 2003). Many of the distinct diencephalic prosomere derivatives that were identified in the rat and mouse brain can be observed in the Mongolian gerbil using Nissl, Acetylcholinesterase (AChE), and Gallyas staining (Figs. 3a–c, 4e, f, 5e, f, 6e, f).

The prethalamus (dp3) is the most rostral diencephalic neuromere, bordering the peduncular hypothalamus (PHy/hp1) (Puelles et al. 2012c, 2021). In the Mongolian gerbil, this dp3 prosomere is found caudal to the TH-positive tracts (SN-striatum, VTA-prefrontal, VTA-limbic) (Figs. 7a–c, 8a, 12a, c, d) (Bilbao et al. 2022; Ferran et al. 2025). For gerbils, the caudal border of dp3 is recognized specifically at the alar plate level, situated rostral to the mammillothalamic tract (mth). The mammillothalamic tract of this species spans from the mammillary body to the anterior thalamic nuclei. Its trajectory begins with an initial rostrocaudal course from the mammillary body towards the basal plate of dp3. At the level of dp3, it undergoes a sharp turn, traversing the dp3 basal plate. Upon reaching the alar-basal plate boundary, it turns caudally, entering the alar plate of dp2. Subsequently, the tract continues its trajectory through dp2, maintaining proximity to the dp2-dp3 boundary, until its termination within the anterior thalamic nuclei, thereby contributing to the Papez circuit (Figs. 3a, c, 4e, f, 5e, f, 6e, f, 8b, c and 13b) (Ferran et al. 2025).

The dp2 prosomere is also known as the thalamic prosomere due to its predominantly thalamic nuclear derivatives arising from the alar plate. Furthermore, the habenular region is also considered an alar derivative of this prosomere (Puelles et al. 2012c; Puelles and Rubenstein 2003). As noted, at the alar plate of dp2, the mth tract identifies its rostral boundary with the prethalamus. However, the caudal limit of dp2, marking its transition to the pretectum (dp1), is defined by the retroflex tract (rf) (Figs. 3a, c, 4f, 5f, 6f, 8b, c, 9b, 13b) (Puelles et al. 2012c; Puelles and Rubenstein 2003; Ferran and Puelles 2024). In Mongolian gerbils, the retroflex tract (rf) originates from the habenular region and initially extends through the alar and basal plates of dp2, near its caudal border. As it approaches the floor plate, the tract undergoes a sharp 90-degree caudal turn, passing through the basal plates of dp1, mdp1, and mdp2 until reaching the hindbrain interpeduncular nucleus (IP) (Figs. 3a, c, 4f, 5f, 6f, 8b, c, 9b, 13b) (Ferran and Puelles 2024; Ferran et al. 2025). The pretectal prosomere (dp1) is the most caudal diencephalic prosomere (Ferran et al. 2007, 2008, 2009). The caudal border of the posterior commissure marks the transition to the midbrain in all vertebrates (the diencephalo-midbrain boundary) (Ferran et al. 2007, 2008, 2009; Morona et al. 2011, 2017; Brożko et al. 2022; Merchan et al. 2011). This boundary is also evident in the Mongolian gerbil brain (Figs. 3c, 4 f, 5f, 6f, 9a, 13a, b). The red parvocellular nucleus (RPC), situated in the basal plate of dp1 (Puelles et al. 2012a, c; Ferran et al. 2025) helps delineate its rostral and caudal limits in the Mongolian gerbil (Figs. 8b, 13b). Tyrosine hydroxylase (TH)-positive processes within the diencephalon proper and secondary prosencephalon (Ferran et al. 2025) also delineate the substantia nigra (SN) and the ventral tegmental area (VTA) in the Mongolian gerbil brain. The distribution of TH-positive neurons includes the basal plate regions of the dp1, dp2, and dp3 prosomeres (Figs. 7a–c, 8a–c, 9a–c, 12a–d,13a, b). A reliable anatomical landmark for delineating TH-positive neurons between the dp1 and dp2 neuromeres is the retroflex tract, which extends into the basal plate, separating the more caudally located dp1 TH neurons from those situated rostrally in dp2 (as shown in Figs. 8b, c and 13b).

### Midbrain of the Mongolian gerbil

The midbrain proneuromere ultimately differentiates into two prosomeres. The midbrain prosomere 1 (mp1) in the Mongolian gerbil brain is also delineated by the tectal gray, superior colliculus, and inferior colliculus in its alar plate, and the III nucleus, its corresponding III nerve, and the red magnocellular nucleus (RMC) in its basal plate between other components (Figs. 2c, d, 3a–c, 4g, h, 5g, h, 6g, h, 7, 8, 9, 13). On the other hand, the midbrain prosomere 2 (mp2) is identified by the presence of preisthmic nuclei (Figs. 2c, d, 3c, 7, 8, 9, 13) (Ferran et al. 2025; Puelles et al. 2012a; Puelles and Hidalgo-Sánchez 2023). As previously described, the diencephalo-midbrain (dp1-mp1) boundary is primarily defined by the caudal border of the posterior commissure, and the red parvocellular nucleus (RPC) on the pretectal side. However, the rostral border of the III nucleus and its corresponding nerve, and the red magnocellular nucleus (RMC) on the midbrain side, can also help identify this rostral midbrain boundary. Importantly, the caudal border of these same structures (III nucleus, nerve, RMC) serves to delineate the mp1-mp2 boundary. These anatomical landmarks are also observable in gerbils (Figs. 2c, d, 3c, 4g, 5g, 6g, 8, 9, 13b). The mp2-r0 boundary, which separates midbrain prosomere 2 from the hindbrain region, is identifiable by several hindbrain derivatives located in rhombomere 0 (r0). Specifically, the IV nucleus, the IV nerve, the decussation of the superior cerebellar peduncle, serve to define its rostral limit with the mp2 prosomere in gerbils (Figs. 2c, d, 3c, 8, 9, 13b) (Ferran et al. 2025). Through these comprehensive analyses, we were able to clearly identify and map the abundant TH-positive processes within the mp1and mp2 prosomeres of gerbils. This precise mapping is critical for understanding the segmental distribution of TH-positive neuronal populations within the SN and VTA (Fig. 12).

### Hindbrain of the Mongolian gerbil

The hindbrain is segmented into thirteen distinct neuromeric units, known as rhombomeres (r0 to r11). Rhombomeres r0, r1r, and r1c collectively form the prepontine region whereas the caudal rhombomeres r2, r3, and r4 define the pontine region. The most caudal rhombomeres r5 and r6 are observed within the retropontine region whereas r7 through r11 constitute the medullary region of the hindbrain (Fig. 2c, d) (Puelles 2018; Watson et al. 2017; Puelles and Hidalgo-Sánchez 2023; Nieuwenhuys and Puelles 2016; Tomas-Roca et al. 2016). In the Mongolian gerbil prepontine, pontine, retropontine and medullary regions present TH-positive processes (Figs. 7, 8, 9, 13). Specifically, rhombomere 0 (r0), which borders the midbrain, is characterized by the presence of the IV nucleus and the decussation of the superior cerebellar peduncle (dcps), but also by SN and VTA TH-positive neurons (Figs. 7, 8, 9, 13). Other nuclei like the interpeduncular nucleus (r0, r1r and r1c) and pontine nuclei (r3,r4) can be clearly observed in the hindbrain region of gerbils (Fig. 3a–c, 4g, h, 5g, h, 6g, h, 7, 8, 9, 13) (Lorente-Canovas et al. 2012; Puelles 2018).

## Discussion

The pattern of TH expression in the brain has been previously used to assess developmental differences between Mongolian gerbils and closely related species (Moon et al. 2007; Wang et al. 2013; Yu et al. 2020). From these studies, it becomes evident that the distinguishing characteristics between species within phylogenetically related families extend beyond mere differences in cell count or density. It implies that more complex factors such as restricted genetic toolset may impose a canalization process (Waddington 1975), thus ensuring a specific neuronal arrangement, which has been maintained since the origin of the Muridae family 45 million years ago (Fig. 1A). In this regard, the prosomeric organization provides an unique conceptual framework for understanding how evolutionary processes shape brain morphology and function by analyzing the comparative neurodevelopment of diverse vertebrate lineages. A compelling illustration of this principle can be observed in the nuanced differences in social behavior among gerbils and other rodent species (e.g. social monogamy shared with prairie voles but not with mice and rats) (McGraw and Young 2010; Gromov 2021).

The primary interneuromeric boundaries in the Mongolian gerbil brain can be delineated at postnatal day one (P1), which are consistent with those observed at P55. For instance, the position of the fornix and catecholaminergic tracts (*ns*, *vta-c*, and *vta-l*) helps define the *hp1-hp2* and *hp1-dp3* boundaries. Other anatomical references like the mammillothalamic tract, retroflex tract, and posterior commissure delineate neuromeric partitions of the secondary prosencephalon. Finally, the oculomotor (III) cranial nucleus and its nerve, the red magnocellular and parvocellular nuclei, and the decussation of the superior cerebellar peduncle are crucial for recognizing midbrain and rostral hindbrain neuromeres.

Our analysis indicates that the distribution of TH-positive neurons and processes in the Mongolian gerbil brain is similar to that found in other rodents and primates, exhibiting a multineuromeric origin spanning from the diencephalon and midbrain, and to the rostral hindbrain (dp1-dp3, mp1, mp2, r0) (Ferran et al. 2025). Furthermore, these TH-positive tracts follow a highly specific and conserved trajectory, providing new insights into the organizational principles of these critical pathways (Fig. 12). To precisely map neuronal distribution, we performed an exhaustive neuromeric characterization, primarily focusing on the forebrain tagma, thereby providing a high-resolution anatomical context for our study (Ferran et al. 2025). This approach is based on the notion that neuromeres are fundamental developmental units of the central nervous system, which provides a more precise anatomical framework that reflects the segmented origin of the brain (Puelles and Rubenstein 2003, 2015; Ferran et al. 2025). Our study identified the same neuromeres in the forebrain of Mongolian gerbils as those described in mice and rats. Specifically, the PHy (hp1) and THy (hp2) for the secondary prosencephalon, pretectum (dp1), thalamus (dp2) and prethalamus (dp3) for the diencephalon proper and mdp1 and mdp2 for the midbrain region (Puelles and Rubenstein 1993, 2003, 2015; Ferran et al. 2015c, 2025; Puelles et al. 2012a, b, c, 2021; Bilbao et al. 2022). In Mongolian gerbils, the fornix exhibits a trajectory identical to that observed in mice and rats, confirming its utility as an anatomical landmark for delineating the boundary between the peduncular hypothalamus (PHy) and terminal hypothalamus (THy) (Ferran et al. 2025; Bilbao et al. 2022; Puelles et al. 2012b). In gerbils, the trajectories of the SN-striatal, VTA-limbic, and VTA-cortical tracts serve as clear indicators of the rostral and caudal boundaries of the PHy as they pass through this neuromere. This pattern aligns with observations made in rats and mice (Bilbao et al. 2022; Ferran et al. 2025). Our analysis also revealed that the interneuromeric boundaries within the diencephalon proper are clearly delineated by the trajectories of specific fiber tracts. The mamillothalamic tract, for instance, coursed within the rostral alar plate of dp2. Similarly, the retroflex tract was found in both the alar and basal plates of dp2, while the posterior commissure identified the alar plate of dp1. These anatomical relationships observed in the Mongolian gerbil brain closely mirror those previously described in mice and rats (Puelles et al. 2012a, c; Ferran and Puelles 2024; Ferran et al. 2008, 2025). In Mongolian gerbils, the third cranial nucleus and its nerve, along with the red parvocellular and magnocellular nuclei, were observed in positions similar to those described in mice and rats. These structures served as landmarks for delineating the neuromeric boundaries (Puelles et al. 2012a, c; Ferran et al. 2025). As observed in mice and rats (Ferran et al. 2025; Puelles and Hidalgo-Sánchez 2023; Lorente-Canovas et al. 2012), we also found that the most rostral rhombomere (r0) in gerbils contains the fourth cranial nucleus and its nerve, the decussation of the superior cerebellar peduncle, and the rostral part of the interpeduncular nucleus. Together, it is conceivable that most of the anatomical components characteristic of these neuromeres (e.g., pallial and subpallial components, hypothalamic, thalamic, pretectal and midbrain derivatives) are identifiable in the Mongolian gerbil brain (Puelles and Rubenstein 1993, 2003, 2015; Ferran et al. 2015c, 2025; Puelles et al. 2012a, b, c, 2021; Bilbao et al. 2022).

The current study also revealed that there are observable anatomical differences between Mongolian gerbils, mice, and rats. When comparing Mongolian gerbils with mice and rats, there are some differences already at P1. For example, the relative size of certain thalamic derivatives, such as the ventroposteromedial nucleus (VPM) and the ventromedial nucleus (VM), appear to differ between gerbils and the other two species. (see Fig. 5e) (Puelles et al. 2012c; Franklin and Paxinos 2008; Paxinos and Watson 2007). Another example is the body of the fornix that upon leaving the proximity of the corpus callosum and following the anterior commissure, it extends deeply into the septal region with only brief contact with the hippocampal commissure (see fx and hc in Fig. 3a). Notably, in gerbils but not in mice and rats, the hippocampal commissure appears to have a broader location, probably reaching the fornix closer to the anterior commissure) (Franklin and Paxinos 2008; Paxinos and Watson 2007). Although comprehensive studies are required, our preliminary mapping of the Mongolian gerbil amygdala indicates potential interspecies variations in the relative sizes of some of its constituent nuclei (e.g., the lateral amygdala [La] and basolateral amygdala [BLA]) when compared with mice and rats (Franklin and Paxinos 2008; Paxinos and Watson 2007). Another example is the trajectory of the mamillothalamic tract. In gerbils, it courses through the basal plate of dp3 before reaching the alar plate of the thalamus (dp2). Studies in mice and rats, however, have primarily described the mamillothalamic tract’s trajectory as passing through both the basal and alar plates of dp2. Further research is needed to determine if these differences represent an interspecies variation and whether the trajectory through the basal plate of dp3 is a common feature among all rodents (Franklin and Paxinos 2008; Paxinos and Watson 2007; Puelles et al. 2012c; Ferran et al. 2025; Bilbao et al. 2022). Similar brain differences between gerbils, mice, and rats persist at P55.

Although future studies are required to extend the analysis to a larger number of rodent species, it could be suggested that the conserved multineuromeric distribution of TH-positive processes within the SN and VTA in rodents could be the result of a restricted genetic toolkit that was present since the origin of the Muridae family 45 million years ago (Fig. 1A). Precisely identifying the ontogenetic origin of brain derivatives in the Mongolian Gerbil using the prosomeric framework will helps determine in further comparative studies which components—from neuromeres to domains and subdomains—are homologous with other mammals or other vertebrates (Puelles and Ferran 2012; Puelles and Rubenstein 2003; Ferran 2017). The origin and the position of the brain derivatives in relation to the prosomeric framework is crucial to establish in further studies whether similar functions arise from homologous brain derivatives or represent convergent evolution from distinct neural regions (Rueda-Alaña et al. 2025). The consistent maintenance of neuromeric boundaries, even with relative differences in component size, points to a common neuromeric identity in rodents. Furthermore, this identity is likely shaped by genetic programs influenced by epigenetic and environmental factors, which allow for a degree of plasticity, notably affecting the size of individual derivatives and their functional connectivity. Collectively, our data corroborates that neuromeres are fundamental developmental and evolutionary modules. Based on the molecular/genetic patterns defining the neuromeric segmentation (Puelles and Rubenstein 1993, 2003, 2015; Ferran et al. 2008, 2015c, 2025; Puelles et al. 2012a, b, c, 2021; Bilbao et al. 2022; Puelles and Hidalgo-Sánchez 2023; Watson et al. 2017), it is conceivable that the emergence of diverse functionality can occur without profound reorganizations of the fundamental structural plan. This highlights their critical role as conserved, yet highly adaptable, units in the evolution of vertebrate nervous systems.

## Data Availability

The entire collection of images of the histological and immunohistochemical processing are not publicly available due to privacy reasons but are available upon request to the corresponding author.

## Abbreviations

3N: Oculomotor complex
3n: Third nerve fibers
A: Amygdala
A13: A13 dopaminergic group
A/B: Alar-Basal boundary
ac: Anterior commissure
aca: Anterior commissure, anterior part
aci: Anterior commissure, intrabulbar part
acp: Anterior commissure, posterior part
Acb: Accumbens nucleus
AOA: Anterior olfactory area
AON: Anterior olfactory nucleus
AP: Anterior pretectal nucleus
At: Acroterminal
BLA: Basolateral amygdala
BMA: Basomedial amygdala
Bstl: Bed nucleus stria terminalis lateral part
Bstm: Bed nucleus stria terminalis medial part
CA1: Field CA1 of the hippocampus
CA2: Field CA2 of the hippocampus
CA3: Field CA3 of the hippocampus
CeA: Central amygdala
Cb: Cerebellum
cc: Corpus callosum
CPu: Caudate-putamen nucleus (striatum)
cp: Cerebral peduncle
Cl: Claustrum
Cx: Cortex
Ct: Caudal terminal
DG: Dentate gyrus
DLG: Dorsal lateral geniculate nucleus
DMH: Dorsomedial hypothalamic nucleus
dp1: Diencephalic prosomere 1
dp2: Diencephalic prosomere 2
dp3: Diencephalic prosomere 3
ec: External capsule
fi: Fimbria of the hippocampus
fx: Fornix
GP: Globus pallidus
HB: Hindbrain
Hb: Habenula
hc: Hippocampal commissure
Hi: Hippocampus
hp1: Hypothalamo-telencephalic prosomere 1
hp2: Hypothalamo-telencephalic prosomere 2
ic: Internal capsule
IC: Inferior colliculus
IG: Indusium griseum
IP: Interpeduncular nucleus
La: Lateral amygdaloid nucleus
LD: Lateral dorsal thalamic nuclei
lfp: Longitudinal fasciculus pons
LG: Lateral geniculate nucleus
LHb: Lateral Habenula
lot: Lateral olfactory tract
LP: Lateral posterior thalamic nucleus
LS: Lateral septal nucleus
LV: Lateral ventricle
M: Mamillary body
MeA: Medial amygdala
MB: Midbrain
MD: Mediodorsal thalamic nuclei
Me: Medullary domain (Medulla Oblongata)
MHb: Medial Habenula
mlf: Medial longitudinal fascicle
mp1: Midbrain prosomere 1
mp2: Midbrain prosomere 2
mtg: Mammillotegmental tract
mth: Mammillothalamic tract
MS: Medial septal nucleus
NH: Neurohypophysis
ns: Nigrostriatal tract
OB: Olfactory bulb
och: Optic chiasm
ot: Optic tract
OV: Olfactory ventricle
P: Pons/Pontine domain
Pn: Pontine nuclei
Pa: Paraventricular nucleus
PAG: Periaqueductal gray
pc: Posterior commissure
PG: Pineal Gland
PG: Pregeniculate nucleus
PHy: Peduncular hypothalamus
Pir: Piriform cortex
Pn: Pontine nuclei
POA: Preoptic area
PrP: Prepontine domain
PT: Pretectum
PTh: Prethalamus
r0: Rhombomere 0
r1r: Rhombomere 1 rostral
r1c: Rhombomere 1 caudal
r2: Rhombomere 2
r3: Rhombomere 3
r4: Rhombomere 4
r5: Rhombomere 5
r6: Rhombomere 6
r7: Rhombomere 7
r8: Rhombomere 8
r9: Rhombomere 9
r10: Rhombomere 10
r11: Rhombomere 11
rf: Retroflex fasciculus (tract)
Rh: Rhombencephalon
RP: Retropontine domain
RMC: Red magnocellular nucleus
RPC: Red parvocellular nucleus
Rt: Reticular nucleus
SC: Superior colliculus
SCh: Suprachiasmatic nucleus
SCo: Spinal cord
Se: Septum
sm: Stria medullaris
SN: Substance nigra
SNc: Substance nigra, compact
SO: Supraoptic nucleus
st: Stria terminalis
St: Striatum
Sub: Submedius thalamic, nucleus
SubG: Subgeniculate nucleus
TG: Tectal gray
Th: Thalamus
THy: Terminal hypothalamus
Tu: Olfactory tubercle
TT: Tenia tecta
VL: Ventrolateral thalamic nucleus
VM: Ventromedial thalamic nucleus
VMH: Ventromedial hypothalamic nucleus
VPL: Ventral posterolateral thalamic nucleus
VP: Ventral pallidum
VTA: Ventral tecmental area
vta-c: Ventral tegmental area cortical tract
vta-l: Ventral tegmental area limbic tract
